# Versatile function of NF-ĸB in inflammation and cancer

**DOI:** 10.1186/s40164-024-00529-z

**Published:** 2024-07-16

**Authors:** Qiang Ma, Shuai Hao, Weilong Hong, Vinay Tergaonkar, Gautam Sethi, Yu Tian, Chenyang Duan

**Affiliations:** 1 Department of Oncology, The Second Affiliated Hospital of Anhui Medical University, Hefei, 230022 P.R. China; 2 Department of Anesthesiology, The Second Affiliated Hospital of Chongqing Medical University, Chongqing, 400010 P.R. China; 3 Research Institute of General Surgery, Jinling Hospital, Affiliated Hospital of Medical School, Nanjing University, Nanjing, 210002 P.R. China; 4 Laboratory of NF-κB Signalling, Institute of Molecular and Cell Biology (IMCB), Agency for Science, Technology and Research (A*STAR), 61 Biopolis Drive, Proteos, Singapore, 138673 Singapore; 5 Department of Pharmacology and NUS Centre for Cancer Research, Yong Loo Lin School of Medicine, National University of Singapore, Singapore, 117600 Singapore; 6 School of Public Health, Benedictine University, Lisle, 60532 USA

**Keywords:** NF-ĸB, Inflammation, Tumor microenvironment, Small molecule inhibitors, Cancer therapy

## Abstract

Nuclear factor-kappaB (NF-ĸB) plays a crucial role in both innate and adaptive immune systems, significantly influencing various physiological processes such as cell proliferation, migration, differentiation, survival, and stemness. The function of NF-ĸB in cancer progression and response to chemotherapy has gained increasing attention. This review highlights the role of NF-ĸB in inflammation control, biological mechanisms, and therapeutic implications in cancer treatment. NF-ĸB is instrumental in altering the release of inflammatory factors such as TNF-α, IL-6, and IL-1β, which are key in the regulation of carcinogenesis. Specifically, in conditions including colitis, NF-ĸB upregulation can intensify inflammation, potentially leading to the development of colorectal cancer. Its pivotal role extends to regulating the tumor microenvironment, impacting components such as macrophages, fibroblasts, T cells, and natural killer cells. This regulation influences tumorigenesis and can dampen anti-tumor immune responses. Additionally, NF-ĸB modulates cell death mechanisms, notably by inhibiting apoptosis and ferroptosis. It also has a dual role in stimulating or suppressing autophagy in various cancers. Beyond these functions, NF-ĸB plays a role in controlling cancer stem cells, fostering angiogenesis, increasing metastatic potential through EMT induction, and reducing tumor cell sensitivity to chemotherapy and radiotherapy. Given its oncogenic capabilities, research has focused on natural products and small molecule compounds that can suppress NF-ĸB, offering promising avenues for cancer therapy.

## Introduction

The nuclear factor-kappaB (NF-ĸB) is an essential molecular regulator of innate and adaptive immune systems. The NF-ĸB is present in the cytoplasm, and shows interactions with its inhibitors, called inhibitors of nuclear factor kappa B (IκBs) The phosphorylation of IκB proteins can lead to their ubiquitylation and proteasomal degradation to induce translocation of NF-ĸB from cytoplasm to nucleus, binding to cognate DNA binding sites in the modulation of gene expression. The interaction of NF-ĸB with IκB proteins (IκBα, IκBβ and IκBε2) in the cytoplasm causes inactivation of NF-ĸB [1]. IκB proteins are known as regulatory proteins characterized by the presence of ankyrin repeats, a motif with 33 amino-acids that causes protein-protein interaction [2]. The function of NF-ĸB in the nucleus as a transcription factor is distinct from its function in the immune system. The stimulation of NF-ĸB occurs in response to infections, free radicals, cytokines, and ultraviolet irradiation, among others, to translocate into the nucleus for binding into DNA sequences affecting biological mechanisms such as apoptosis and cell growth. The survival and stimulation of lymphocytes can be mediated by NF-ĸB, and it also participates in the regulation of immune reactions. The activation of NF-ĸB has been associated with a number of inflammatory conditions, including arthritis, inflammatory bowel disease, multiple sclerosis, and asthma, among others [3].

The NF-ĸB family has been comprised of the proteins including RELA (p65), NF-κB1 (p50; p105), NF-κB2 (p52; p100), c-REL and RELB [1, 2]. The proteins of the NF-ĸB family have a conserved structure comprising of an amino-terminal region consisting of around 300 amino-acids with dimerization, nuclear-localization, and DNA-binding domains. Moreover, c-REL, RELB, and RELA possess carboxy-terminal non-homologous transactivation domain participating in the stimulation of transcription from NF-ĸB-binding sites in the targeted genes. Noteworthy, other REL proteins, such as p50 homodimers, lack a transactivation domain, but their function in suppressing transcription has been revealed due to their capacity to bind to NF-ĸB consensus sites in the DNA [4]. The proteolytic processing of precursors p105 and p100 protein leads to the formation of p50 and p52 proteins, respectively [5]. Moreover, the members of the NF-ĸB family have the ability to form homodimers and heterodimers with each other, except for RELB. The major stimulated form of NF-ĸB is its heterodimerization with the p65 subunit and also, association with the p50 or p52 subunit. Notably, the ubiquitous expression of p50 and p65 is observed in different cell types, while RELB expression is limited to a number of specific tissues, including the thymus, lymph nodes, and Peyer’s patches. Moreover, c-REL is expressed in hematopoietic cells and lymphocytes. NF-ĸB participates in the transcription regulation of RELB, c-REL, and p105 [1, 2].

**Fig. 1:**
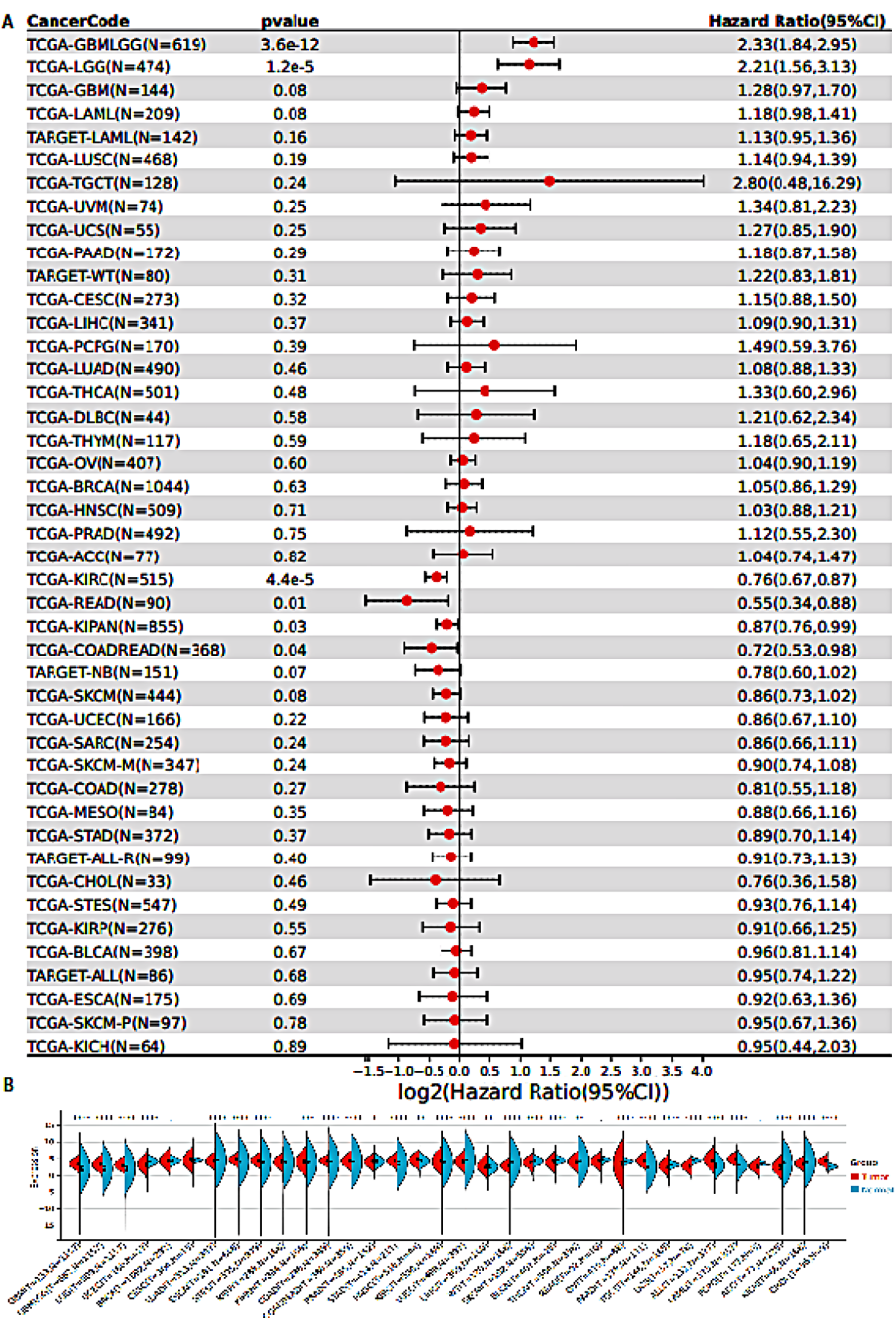
The survival and expression analysis of NF-ĸB in human cancers. **A**) Forest plot highlights the association between NF-ĸB and the overall survival of cancer patients. The upregulation of NF-ĸB has significant association with prognosis of glioma patients (poor prognosis); **B**) The Violin plot compares the expression of NF-ĸB in tumor and normal cells in different human cancers. (Created from TCGA database) (https://www.cancer.gov/ccg/research/genome-sequencing/tcga)

Moreover, ankyrin repeats can be found in p100 and p105 NF-κB proteins. Interestingly, the precursor proteins can be cleaved in the domain containing ankyrin repeats through the proteolytic mechanism to be degraded. BCL-3 is an unusual member of the IκB family that it can develop homodimers with p50 and p52 to stimulate the expression of NF-κB-related genes. Therefore, the function of BCL-3 is in contrast to other IκB proteins [4, 6]. The members of the NF-κB family share an amino-terminal REL homology domain known as RHD [7]. In the canonical pathway of NF-κB, RelA and p50 heterodimers mediate the transcription of the targeted genes, whereas the non-canonical pathway of NF-κB is mediated by the heterodimer formation of RelB and p52 [8, 9]. IκB proteins stimulate the sequestration of RelA and p50 in the cytoplasm. The typical IκB proteins are categorized into three groups, including IκBα, IκBβ, and IκBε [10–12], p100 and p105 as precursor proteins [13], and atypical IκB proteins comprised of IκBζ, BCL-3, and IκBNS [11, 14, 15]. The IKKs stimulate the phosphorylation of IκB to induce the canonical NF-κB axis. IKK has two subunits, including IKKα (also known as IKK1) and IKKβ (also known as IKK2) as catalytic factors and IKKγ as the regulatory subunit [16]. Figure 1 demonstrates the expression analysis of NF-ĸB in various human cancers and its association with the survival rate of patients. The forest plot (Fig. 1A) evaluates the association of NF-ĸB with the overall survival of cancer patients that a HR higher than 1 suggests the association of NF-ĸB with poor prognosis, while a HR less than 1 recommends the association of NF-ĸB with favourable prognosis in cancer patients. The HR equal to 1 does not have impact. Among the cancers, NF-ĸB shows association with poor prognosis in glioma (LGG and GBMLGG), while it demonstrates favourable association with prognosis in kidney renal cell carcinoma (KIRC) and rectum adenocarcinoma (READ). The Biolin plot (Fig. 1B) demonstrates the changes in the expression level of NF-ĸB in cancers and its comparison with the normal tissues.

**Fig. 2:**
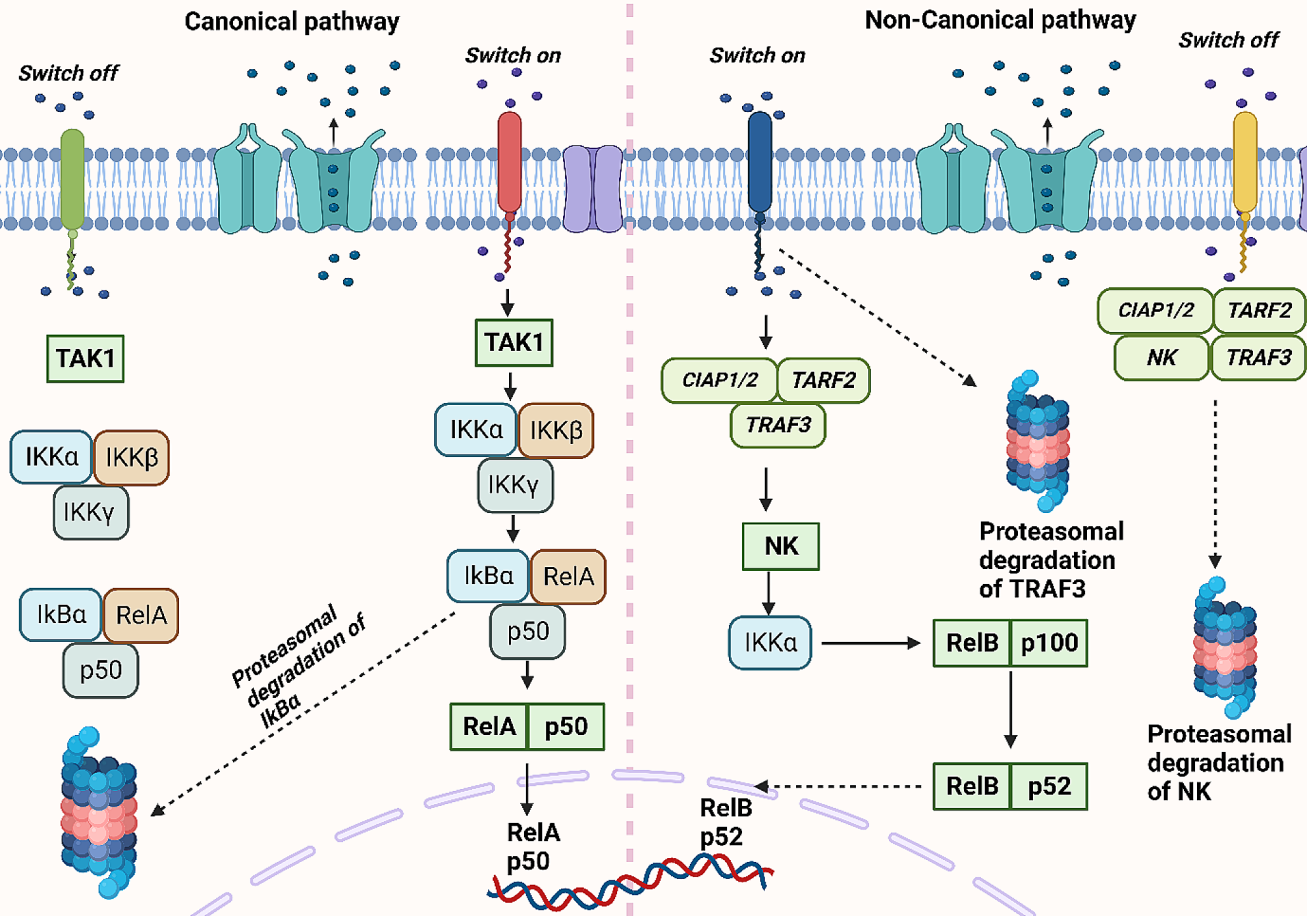
The NF-κB signaling in cells. There are two pathways for NF-κB, including canonical and non-canonical. In the canonical pathway, when there are stimuli such as TNF-α, IL-1β and LPS, the stimulation of NF-κB occurs through upregulation of TAK1 to increase the activity of IKKs for increasing proteasomal degradation of IkBα to further facilitate the nuclear transport of RelA and p53 for the regulation of genes. In the non-canonical pathway, the presence of LTβ, CD40L, and BAFF can stimulate it to promote proteasomal degradation of TRAF3 for stimulation of RelB and p52 transfer to the nucleus to regulate the expression level of genes (Created by Biorender.com)

In response to pro-inflammatory cytokines and different microbial products, IKKβ mediates the canonical pathway of NF-κB, whereas the non-canonical pathway of NF-κB is controlled by IKKα [17–19]. As a catalytic factor, NF-κB Essential Modulator (NEMO) is vital for the canonical pathway of NF-κB. The interaction of NEMO with IKKs is performed through N-terminal, whereas NEMO interacts with upstream signaling adaptors through C-terminal. The oligomerization of IKKα/β/γ stimulates the IKKβ kinase activity [16, 17, 20]. In order to relieve the NF-κB dimers from the cytoplasm suppressors, IKKβ facilitates the IκB protein phosphorylation to accelerate the ubiquitination and degradation of IκBs [21, 22]. Then, the transport of NF-κB dimers to the nucleus occurs to modulate the expression level of target genes [14, 21, 23]. The stimulation of the canonical NF-κB axis in innate and adaptive immune cells has been observed as a response to innate PRRs, TCR, BCR, and pro-inflammatory cytokine receptors [9, 23, 24]. Moreover, the induction of the IKK complex is a result of certain adaptor molecules, ubiquitin ligases, and protein kinases [13, 16]. For the non-canonical pathway of NF-κB, RelB/p52 heterodimers are stimulated as essential transcription factors. The precursor of the p52 protein is p100, which impairs the transfer of RelB to the nucleus. Moreover, when proteolysis of p100 occurs, it promotes p52 production and releases RelB to cause the formation of RelB/p52 dimer for transporting into the nucleus. Therefore, the non-canonical NF-κB axis is modulated by p100 and is also controlled by the NIK-IKKα complex [25–27]. Figure 2 schematically represents the NF-κB and its related mechanisms and pathways.

The increasing evidence has highlighted that NF-κB undergoes sustained activation in the tumor cells. There are several reasons for the induction of the NF-κB axis, including the presence of cytokines, growth factors, and tyrosine kinases. Notably, the kind of cytokine affecting the NF-κB axis determines the function of this pathway. For instance, the stimulation of the NF-κB axis by IL-1 can change the activity of NF-κB from anti-apoptotic to pro-apoptotic [28]. The cytokines and growth factors are able to mediate autocrine or paracrine induction of NF-κB axis [29]. Another reason for the stimulation of NF-κB is the phosphorylation of IκBs by the IKK complex, mediating the degradation by the proteasome pathway to hyperactivate NF-κB [30]. The activation of NF-κB may also be induced by the upregulation of VEGFR, IGFR, and TNF family members. Furthermore, the upregulation of Ras/MAPK and PI3K/Akt is responsible for the activation of NF-κB [31]. The chromatin remodeling is another factor in the activation of NF-κB. Notably, oxidative stress results in the acetylation by histone acetyltransferase, which mediates the uncoiling of DNA and enhances accessibility to the transcription factor binding sites, thereby inducing the NF-κB axis for the generation of pro-inflammatory factors [32]. The activation of the NF-κB axis can also occur as a result of epigenetic changes, especially the upregulation or downregulation of non-coding RNAs (miRNAs, lncRNAs, and circRNAs) to change the process of tumorigenesis. Finally, the presence of feedback loops ensures the activation of NF-κB. For instance, FGL1-mediated NF-κB (p65) activation upregulates STAT3 expression, and due to the presence of a feedback loop, STAT3 increases FGL1 expression to upregulate NF-κB (p65) [33].

The current paper provides a comprehensive discussion of NF-κB function for the regulation of tumorigenesis and inflammation in cancers. Since inflammation has been linked to the transformation of inflammatory diseases such as colitis to cancer, the interaction of NF-κB and inflammation will be discussed. Moreover, NF-κB regulates the release of cytokines and inflammatory factors, including interleukins (ILs) and tumor necrosis factor-α (TNF-α) during cancer progression. NF-κB is able to modulate vital biological mechanisms in tumors, such as cell death pathways (apoptosis, autophagy, and ferroptosis), tumor metabolism, cancer stem cells, angiogenesis, and anoikis. Moreover, NF-κB can modulate the important hallmarks of cancers, including proliferation, metastasis, and therapy resistance. Since NF-κB has been considered a key player in cancer progression, its regulation by epigenetic factors such as non-coding RNAs is discussed. Moreover, the application of natural products and, small molecules for the suppression of NF-κB in the treatment of cancers is described in the current review.

## NF-ĸB in the regulation of inflammation in cancer

**Fig. 3:**
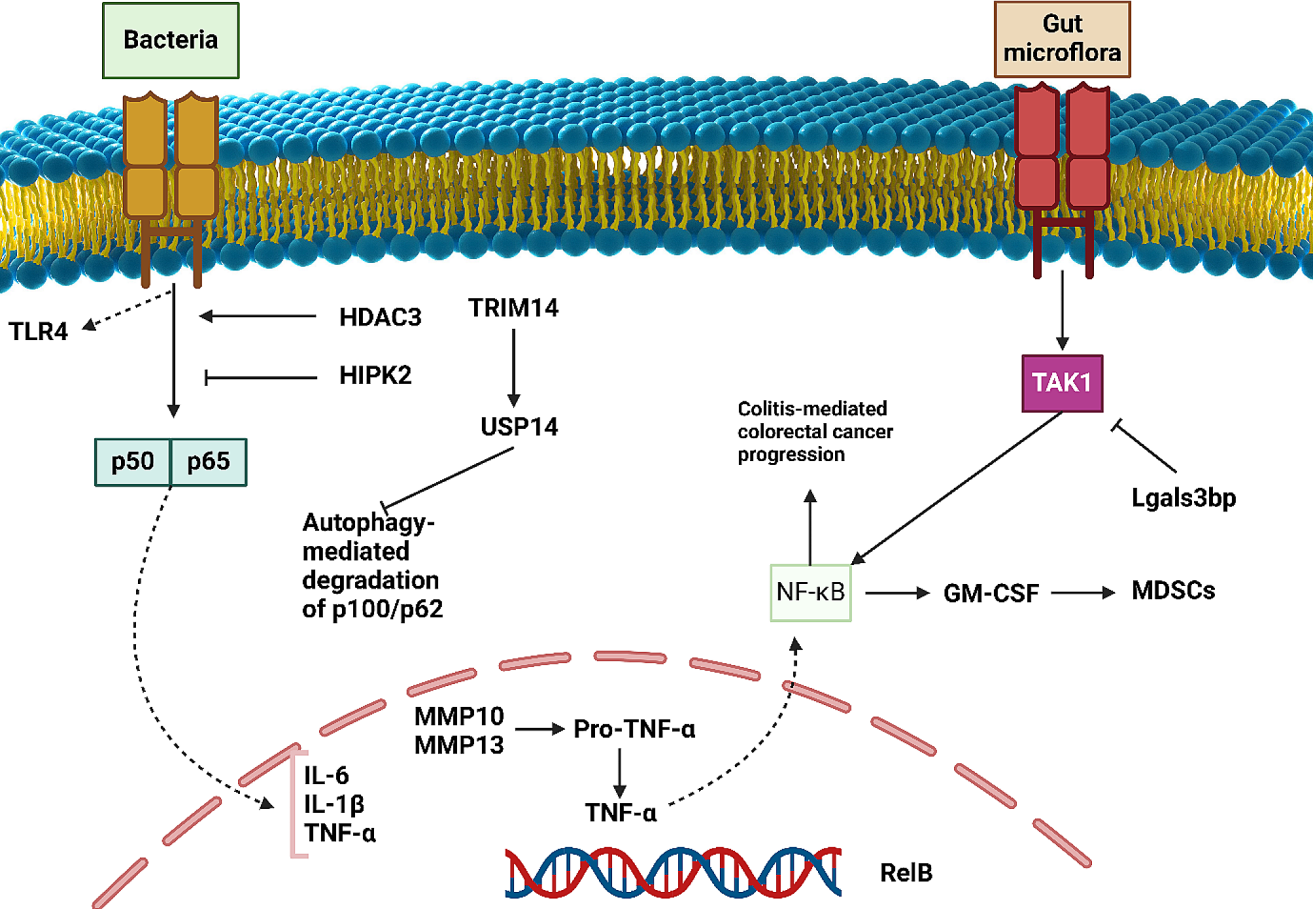
NF-κB, inflammation, and cancer progression. Based on the evidence, NF-κB can cause inflammation to promote tumorigenesis, and in turn, inflammation can cause stimulation of NF-κB-mediated carcinogenesis. Therefore, there is a positive feedback loop between NF-κB and inflammation in the regulation of cancer progression. Moreover, the presence of exogenous insults such as bacterial infections and gut microflora can stimulate the TLR4 receptor to induce the NF-κB axis. The non-canonical pathway of NF-κB also interacts with BCL-3 in promoting cancer progression. Moreover, when TRIM14 is induced, it suppresses the degradation of p100/p52 by autophagy to maintain the function of NF-κB in cancer progression (Created by Biorender.com)

Cancer is a dynamic and complex disease occurring in various steps in which each step has a significant association with inflammation [34]. This connection can be observed from the initial steps of tumorigenesis and neoplastic transformation, extending to proliferation and metastasis [35]. Therefore, the interaction between inflammation and cancer is not surprising. A number of immune-induced inflammatory disorders are related to the development of cancer [36]. For instance, the presence of inflammatory bowel disease can cause colorectal cancer [37]. Moreover, chronic obstructive pulmonary disease (COPD) has been shown to increase lung cancer [38–40]. During COPD, hypoxia is induced, which causes the activation of transcription factors at low oxygen levels to impair apoptosis [41]. Moreover, the presence of COPD and inflammation can reduce the potential of the lungs to expel toxins, enhancing the risk of lung cancer development [42]. Notably, there is a connection between chronic psoriasis and different kinds of cancers, including lymphomas and skin tumors [43]. The presence of chronic inflammation from bacteria and viruses as infectious diseases can cause cancer [44]. Therefore, there is a strong connection between inflammation and cancer. Given the intricate role of NF-κB in modulating inflammation-related pathways and its implications in cancer progression, the regulatory mechanisms controlling NF-κB activity become crucial in understanding its dual role in health and disease. Specifically, the process of IκB degradation illustrates how NF-κB activity is finely tuned in response to inflammatory stimuli, which can either protect against or contribute to the pathogenesis of cancer, depending on the cellular context and the nature of the inflammatory response. The interaction of NF-κB and inflammation can change the process of cancer progression. To this end, understanding how NF-κB regulates inflammation can broaden the knowledge towards the process of carcinogenesis. The pattern-recognition receptors stimulate certain transcriptional nodes and modules through the modulation of signaling cascades [45]. Although NF-κB has a significant association with the regulation of inflammation, other kinds of molecular pathways, such as JAK/STAT or MAPK, can also control inflammation [46, 47]. The regulation of target genes by NF-κB can orchestrate and control a number of essential biological mechanisms, including inflammatory mediators, cell growth and viability, differentiation of T cells, and others. Among them, the dysregulation of NF-κB has been correlated with the development of inflammatory and autoimmune diseases as well as cancer [48, 49]. IκB degradation is a crucial event in the activation of the NF-κB pathway, primarily mediated by its K-48-linked ubiquitination. This post-translational modification targets IκB for proteasomal degradation, thereby allowing NF-κB to translocate to the nucleus and initiate transcription. The ubiquitin-proteasome system (UPS) selectively degrades IκB through the attachment of K-48 linked ubiquitin chains, a process catalyzed by the SCF^β-TrCP E3 ubiquitin ligase following phosphorylation of IκB by the IKK complex [50]. This ubiquitination is not merely a signal for degradation but also a regulatory mechanism that maintains the balance of NF-κB activity within the cell, critical for cellular responses to inflammation and immune challenges. Interestingly, the stimulation of NF-κB is restricted by itself through overexpression of IκBα and other NF-κB inhibitors. In order to approve this hypothesis, an experiment has been performed in mice, and the results advocate the fact that κB enhancer site mutation in IκBα promoter predisposes to the development of pathological events, including autoimmunity, dysregulation of T cell development and hypersensitivity to the endotoxic shock, because of the generation of inflammatory factors [51]. The other members of the IκB family with different affinities for NF-κB targets also confirm the presence of this negative feedback. As a result of sustained induction of IKK and stimulus-mediated upregulation of IκBɛ, observations provide a distinct function for IκBα in the control of B cell proliferation and survival [52]. The pathogen-associated molecular patterns are believed to contribute to the expression of IRAK-M kinase for suppressing the NF-κB axis and preventing its interaction with downstream targets [53]. Figure 3 schematically demonstrates the association between NF-κB, inflammation, and cancer progression.

## NF-ĸB and tumor microenvironment components

### NF-ĸB and tumor-associated macrophages

The tumor microenvironment (TME) is a complex environment capable of shaping and determining the progression of tumors and their response to immunotherapy [54] and has been comprised of the different kinds of cells including myeloid cells [55] and dendritic cells [56], among others. Macrophages are considered as immunocytes with broad-spectrum functions ranging from tissue homeostasis, pathogen attack defense, and wound healing acceleration [57]. Therefore, macrophages regulate both innate and adaptive immune responses [58]. However, such macrophages can infiltrate into the TME, called tumor-associated macrophages (TAMs). The hallmarks of cancer, including proliferation, angiogenesis, invasion, drug resistance, and immune reactions, are regulated by TAMs [59–61]. The advances in biology have identified new subtypes of TMAs such as CD86^+^ TAMs regulating carcinogenesis [62]. TAMs mainly accumulate in the leading edge and vascular regions, whereas the rest of TAMs accumulate in the abluminal side of vessels [63, 64]. TAMs are present in two phenotypes, including M1 and M2. The current ideas demonstrate the versatile function of NF-κB axis in the regulation of TAM infiltration in the TME. Moreover, there is evidence that the phagocytosis function of TMAs is also regulated by NF-κB in cancer. Therefore, broadening the knowledge towards the regulation of TAMs by NF-κB can provide new insights into the development of therapeutics for cancer. The pro-oncogene function of TAMs can be kept by the NF-κB. The predominant expression of Macrophage-inducible C-type lectin (Mincle) is observed in TAMs. Moreover, the function of Mincle in the regulation of NF-κB in TAMs is independent of TLR4. Mincle stimulates the NF-κB axis in an effort to increase the function of M2-like TAMs in lung cancer progression [65]. In addition to regulation of TMA function in tumorigenesis, the NF-κB axis can also participate in the recruitment of macrophages in cancer progression. Upon TAp73 deficiency, stimulation of the NF-κB axis occurs to promote CCL2 secretion for recruiting monocytes and macrophages. The loss of TAp73 accelerates the accumulation of TAMs with pro-carcinogenic function with upregulation of CD206 and CD204 [66]. The impact of CCL2 on the recruitment of macrophages in tumorigenesis for lung cancer has also been highlighted. Neddylation suppression reduces the function of CRLs to increase IκBα-mediated NF-κB inhibition. However, the neddylation pathway in lung cancer can stimulate the NF-κB axis to promote the secretion of CCL2 for increasing infiltration and accumulation of TAMs to suppress lung cancer progression and mediate poor prognosis [67]. Since M2 polarized macrophages demonstrate upregulation of NF-κB, there is a notion that suppression of NF-κB may help in macrophage reprogramming in cancer therapy. DRD2 interacts with β-arrestin2, DDX5, and eEF1A2 to suppress the NF-κB axis, leading to M1 polarization of macrophages and increasing pyroptosis in breast cancer [68].

### NF-ĸB and cancer-associated fibroblasts

Another member of TME is cancer-associated fibroblasts (CAFs) that participate in various hallmarks of cancer, including growth, metastasis, drug resistance, and immune evasion [69–71]. The proliferation of tumor cells after radiotherapy increases by CAFs and also induces EMT through upregulation of VEGF and FGF [72, 73]. The interaction of CAFs with NF-κB axis in cancer can change the process of tumorigenesis. For exerting many of the carcinogenic functions, CAFs stimulate the NF-κB axis. The overexpression of NF-κB in fibroblasts and secretion of IL-6 was documented by Nakshatri and colleagues in 1998 [74]. Then, in 2009, Jacks and colleagues demonstrated that p53 and G12D loss can upregulate NF-κB expression in fibroblasts [75]. Therefore, these results provide the idea that CAFs demonstrate the expression of NF-κB. The CAFs in the TME secrete IL-8 that after binding to IL-8R, stimulates NF-κB. Upon nuclear translocation of NF-κB, it increases DNA damage repair to prevent cell death, accelerating growth and radioresistance [76]. However, the NF-κB function in the development of radioresistance requires the PARP-1 role [77]. It appears that the secretion of inflammatory factors by CAFs is the most prominent way to increase the progression of tumor cells through the upregulation of NF-κB. IL-6 secreted by CAFs can bind to IL-6R on the surface of tumor cells. Then, it enhances the phosphorylation of STAT3 to mediate the secretion of osteopontin from tumor cells. Then, osteopontin binds to the integrin αVβ3 receptor to induce NF-κB nuclear translocation for increasing levels of ICAM-1, uPA, MMP-9, and MMP-2 to facilitate growth and invasion of head and neck cancer cells [78]. In addition to the modulatory impact of CAFs on the NF-κB axis for tumorigenesis regulation, there is also evidence that the NF-κB axis can change the function of CAFs in cancer. Regarding the presence of epigenetic changes in pancreatic cancer, circCUL2 sponges miR-203 to increase MyD88 expression at the mRNA level. Then, MyD88 stimulates the NF-κB axis to promote the production of IL-6. Then, IL-6 binds to IL-6R on the surface of cancer cells to induce STAT3 axis for cancer progression. The aggressive pancreatic tumor cells secrete IL-6 to induce the inflammatory phenotype in CAFs [79].

### NF-ĸB, lymphocytes and T cells

The crosstalk of NF-κB with TAMs and CAFs revealed the function of NF-κB as a pro-oncogene. However, in evaluating the interaction of NF-κB with T cells, the idea is a little complicated, and it is explained that in some cases, the overexpression of NF-κB can improve the anti-cancer function of T cells and aid in cancer immunotherapy. The first aspect is the regulation of lymphocyte function by NF-κB. The role of cytotoxic lymphocytes against cancer or viral infections is suppressed by the function of NF-κB in the upregulation of Snail to suppress RKIP [80]. Such regulation of lymphocytes by the NF-κB axis can change the microenvironment to favor cancer progression. In a hypothesis, it was tested if NF-κB has a function in linking COPD to lung cancer. The chronic stimulation of NF-κB can promote levels of M2 polarized macrophages and Foxp3 regulatory T lymphocytes to promote the development of lung cancer from COPD [81]. The upregulation of Fascin in lymphocytes can increase aggressive behavior and migration potential. LMP1 promotes levels of Fascin in lymphocytes, and suppression of NF-κB using inhibitors downregulates Fascin levels in lymphocytes [82]. However, stimulation of the NF-κB axis in some cases can accelerate anti-cancer immunity. Salvia miltiorrhiza polysaccharide has the ability to increase levels of TLRs, including TLR1, TLR2, and TLR4, to stimulate MAPK and NF-κB axis for stimulation of T lymphocytes [83]. The regulation of T cells by NF-κB can also change the response to chemotherapy. The CCL20 secretion by colorectal cancer cells can induce 5-fluorouracil resistance through NF-κB upregulation through stimulation of regulatory T cells (Treg cells) [84]. The expression of NF-κB can also mediate sex differences in cancer. Androgen signaling stimulates androgen receptors to increase USP18 expression. Then, it suppressed TAK1 phosphorylation to promote NF-κB expression to disrupt the function of T cells. Testosterone synthesis reduction or castration can increase the anti-cancer function of T cells and promote the potential of anti-PD-1 immunotherapy [85]. However, upregulation of NF-κB and p300/CBP can increase MHC-I antigen presentation, showing that stimulation of NF-κB axis can participate in cancer immunotherapy [86]. The anti-cancer function of NF-κB is due to its ability to recruit CD8 + T cells. STK3, as a suppressor of ovarian cancer, stimulates the NF-κB axis to increase the recruitment and migration of CD8 + T cells in cancer therapy [87]. On the other hand, a number of T cells, such as Th17, can secrete IL-6 and TNF-α to stimulate STAT3 and NF-κB in accelerating the proliferation and progression of colorectal cancer [88].

### NF-ĸB and natural killer cells

The natural killer (NK) cells are the key barriers against cancers, and they directly interact with malignant cells for their killing through proteolytic granzymes and secretion of cytokines such as IFN-γ, MIPs, IL-8, IL-10, and TNF-α. Moreover, these cytokines can participate in the recruitment of other immune cells, such as myeloid and Th1 cells, to potentiate cancer immunotherapy [89, 90]. Moreau and colleagues demonstrated that NF-κB can be induced in NK cells through the function of soluble HLA-G [91]. In the treatment of cervical cancer, lirilumab and avelumab participate in the increase of NK cell-induced lysis of tumor cells. Moreover, they enhance the infiltration of NK cells and promote cytolysis. These drugs also promote NK cell NF-κB disinhibition in cancer immunotherapy [92]. The secretion of IFN-γ by NK cells can improve the potential for cancer suppression. TAARD is a synthetic derivative of diphyllin that promotes levels of TLR1 in NF-κB phosphorylation while it increases TLR3 expression in STAT3 phosphorylation to promote IFN-γ levels in potentiating function of NK cells in cancer therapy [93]. In a hypothesis, it was mentioned that the downregulation of HSF1, SP1, and NF-κB by NZ28 can reduce the NK-activating ligands MICA/B on the cancer cells [94]. Split hand and foot malformation 1 (SHFM1) participates in the induction of the NF-κB axis by mediating nuclear translocation of p65. The loss of SHFM1 disrupts the NF-κB axis and promotes NK potential in tumor suppression [95].

### NF-κB and dendritic cells

**Fig. 4:**
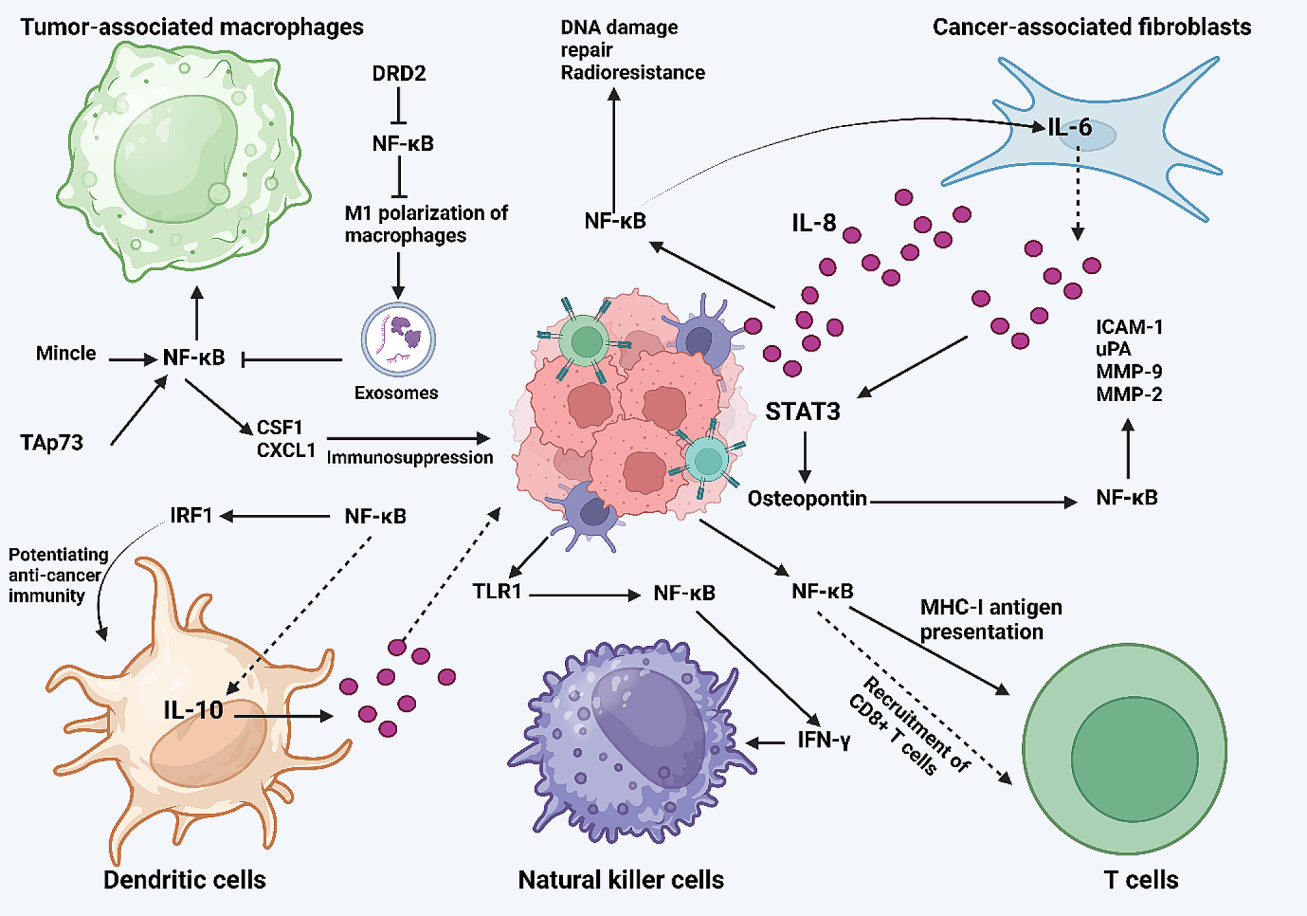
The NF-ĸB axis and tumor microenvironment components. The TAMs and CAFs are primary regulators of cancer progression through interaction with NF-ĸB. The secretion of IL-8 from CAFs can induce NF-ĸB to increase DNA damage repair and radioresistance. Moreover, activated NF-ĸB forces CAFs to secrete IL-6 for stimulation of the STAT3/osteopontin axis, causing NF-ĸB upregulation and acceleration in the metastasis and proliferation of cancer cells. The upregulation of NF-ĸB by Mincle and Tap73 can enhance the recruitment and M2 polarization of macrophages. Moreover, NF-ĸB downregulation by DRD2 increases M1 polarization of macrophages to secrete exosomes for inhibition of NF-ĸB. The secretion of CSF1 and CXCL1 as a result of NF-ĸB activation can cause immunosuppression. NF-ĸB promotes the recruitment of CD8 + T cells and MHC-I antigen presentation. Moreover, TLR1 upregulation activates NF-ĸB to release IFN-γ in the stimulation of NK cells. Finally, IRF1 upregulation by NF-ĸB can increase anti-cancer immunity. Moreover, NF-ĸB accelerates the release of IL-10 from DC cells to affect cancer progression (Created by Biorender.com)

Another component of the immune system is dendritic cells (DCs) that can connect innate and adaptive immune systems. In the innate immune system, the DCs participate in the identification of PAMPs and DAMPs through the pattern-recognition receptors [96]. The function and properties of DCs are regulated by NF-κB axis. The demethylation of NF-κB/p65 can be induced by vitamin C and promotes immune-related genes during the maturation of DC. Moreover, NF-κB p65 interacts with TET2 in the regulation of vitamin C-induced alterations. Moreover, vitamin C enhances the generation of TNFβ in DC cells through the NF-κB axis [97]. It appears that the function of the NF-κB axis can accelerate the immune responses mediated by DCs in cancer therapy. Hence, therapeutic upregulation of NF-κB in this case is suggested. NF-κB promotes IRF1 expression to induce programming of cDC1 in accelerating anti-cancer immunity [98]. Furthermore, the release of IL-10 from DCs is mediated by the function of NF-κB p65 [99]. Figure 4 shows the association of NF-ĸB with tumor microenvironment components.

## Biological associations of NF-ĸB

### NF-ĸB and autophagy

Since Christian de Duve introduced autophagy in 1963, research has made significant strides in understanding its biological aspects and exploring its clinical applications [100, 101]. Among the different pathological events in which autophagy is involved in their pathogenesis and progression, cancer has been in the spotlight [102]. Autophagy has been considered as a suppressor of cancer. However, other studies revealed that stimulation of autophagy flux can accelerate the survival and proliferation of cancer cells [103, 104]. As a result, a question arises regarding the utilization of autophagy in cancer: should we induce or suppress autophagy in cancer? In premalignant lesions, the stimulation of autophagy has been proposed for cancer suppression [105]. In advanced and metastatic tumors, a combination of stimulation and suppression of autophagy is used for therapeutic purposes [103, 106, 107]. Until now, a high number of autophagy regulators have been introduced and from a molecular standpoint, AMPK, Beclin-1, PI3K/Akt/mTOR, ATGs, and non-coding RNA are the most prominent regulators of autophagy. Based on recent advances, autophagy regulates various hallmarks of cancer, including growth, metastasis, and chemoresistance [108, 109]. The interaction of autophagy and NF-ĸB has provided new insights for the treatment of cancer. NF-ĸB may be involved in the induction of pro-survival autophagy to increase cancer progression. Moreover, autophagy can regulate the NF-ĸB axis by targeting the related regulators. Therefore, the interaction of autophagy and NF-ĸB is mutual, and dysregulation of both can fuel tumorigenesis. At first, the current idea is that NF-ĸB stimulates protective autophagy to increase 5-fluorouracil resistance in cancer. For stimulation of the NF-ĸB axis, CD13 is vital. Ubenimex has been introduced as a suppressor of autophagy to increase drug sensitivity in gastric cancer. Ubenimex downregulates CD13 expression to interfere with the EMP3/FAK/NF-κB axis, suppressing autophagy and increasing 5-fluorouracil sensitivity in gastric tumors [110]. However, since autophagy function in cancer is complicated, it may create some confusion in understanding the interaction between NF-ĸB and autophagy in cancer. For instance, based on the findings of a previous study, NF-ĸB induces autophagy in cancer drug resistance. However, what happens if NF-ĸB induces autophagy with toxic and lethal function? In this case, NF-ĸB-mediated autophagy reduces tumorigenesis. This idea has been evaluated in hepatocarcinoma that autophagy induction can impair carcinogenesis. Bisindolylmaleimide alkaloid BMA-155Cl promotes the levels of NF-ĸB, Beclin-1, p53, and Bax to induce both apoptosis and autophagy in hepatocarcinoma suppression. As a result, NF-ĸB-induced autophagy impairs cancer progression [111]. However, the interaction of autophagy and NF-ĸB is not only related to the interaction of molecular pathways and sometimes, the receptors on the surface of cancer cells can provide the interaction of autophagy and NF-ĸB axis. An example is the function of the NOP receptor. The upregulation of E2F1 in the nucleus can induce the NOP receptor to increase the nuclear transfer of the p65/p50 complex. Then, the p65/p50 complex promotes LC3B expression and downregulates p62 to induce autophagy to facilitate the proliferation of cancer [112]. Moreover, NF-ĸB can function as a mediator between endoplasmic reticulum (ER) stress and autophagy. ER stress induces the NF-ĸB axis to induce autophagy for the suppression of cervical cancer [113]. Therefore, it can be concluded that NF-ĸB is an upstream inducer of autophagy in cancer [114].

### NF-ĸB and apoptosis

In the recent years, the regulatory mechanisms of cell death pathways have been of interest and therefore, the studies have focused on understanding the various cell death pathways including pyroptosis, ferroptosis, apoptosis and others in human cancers [115–117]. Apoptosis is considered the most studied cell death, among others. Cell death mechanisms are conserved processes among species, and their study was started from lower organisms, including C. *elegans*, by Robert Horvitz, who obtained the Noble Prize in Physiology or Medicine in 2002 [118–120]. The early knowledge and understanding of cancer pathogenesis comes from the viral and cellular oncogenes [121], cellular growth, and transformation. After the identification of DNA fragmentation upon glucocorticoid exposure [122] and also in the tumor cells that underwent chemotherapy [123], the stimulation of apoptosis was considered a promising idea in cancer therapy. Apoptosis has intrinsic and extrinsic pathways involving mitochondria and death receptors, respectively. The regulation of apoptosis by NF-κB has been observed in various human cancers. The suppression of FGFR4 and EZH2 can stimulate apoptosis through YAP downregulation in hepatocellular carcinoma. Notably, the accumulation of EZH2 in tumor cells is performed by the non-canonical pathway of NF-κB [124]. Therefore, the non-canonical pathway of NF-κB can be considered as a mechanism in apoptosis suppression. The various cell lines use different pathways of NF-κB for apoptosis regulation. For induction of apoptosis in leukemia, dimethyl fumarate suppresses the NF-κB axis; notably, this compound suppresses both canonical and non-canonical pathways of NF-κB in MT-2 cells, while it only suppresses the non-canonical pathway of NF-κB to induce apoptosis in MT-1 cells [125]. Since the apoptosis induction is based on mitochondrial dysfunction, the changes in the homeostasis of mitochondria can also alter the NF-κB axis. The increase in mitochondrial ROS can stimulate MAPK/ERK and NF-κB pathways to mediate apoptosis [126]. Since ROS levels can activate NF-κB, the cautions should be considered for the application of compounds that can affect ROS levels and how they direct NF-κB for tumorigenesis.

### NF-ĸB and ferroptosis

Oxidation of lipids can induce ferroptosis, a new kind of cell death that, despite evaluation in mammalian systems for the first time [127], is also observed in other evolutionary remote species, including plants, protozoa, and fungi [128–130]. The presence of environmental stresses and/or intra/inter-cellular signaling can change the cellular metabolism to cause peroxidation of lipids for the induction of ferroptosis [131]. Recently, therapeutic induction of ferroptosis has obtained much attention in terms of cancer suppression. However, an effective therapeutic should be based on the underlying mechanisms. Ferroptosis is regulated by the NF-ĸB axis in tumor cells. The changes in NF-ĸB can increase ferroptosis sensitivity in cancer. SHARPIN has been introduced as an inducer of ferroptosis in cancer. The function of SHARPIN in the regulation of ferroptosis in sarcoma is based on the NF-ĸB control [132]. The interaction of NF-ĸB and ferroptosis can determine the response to chemotherapy. The application of isoliquiritin can stimulate ferroptosis in breast tumors through NF-ĸB suppression to enhance doxorubicin sensitivity. Moreover, isoliquiritin reduces levels of GSH, GPX4, and xCT, while it enhances Fe^2+^, ROS, and MDA to accelerate ferroptosis in breast cancer [133]. Therefore, NF-ĸB inhibition can accelerate ferroptosis in human cancers. For inhibition of ferroptosis in cancer, NF-ĸB promotes levels of SLC7A11. Since SLC7A11 is a suppressor of ferroptosis, its upregulation by NF-ĸB induces ferroptosis resistance. Aspirin suppresses the NF-ĸB axis to downregulate SLC7A11 to facilitate ferroptosis in hepatocellular carcinoma [134]. Since the function of NF-ĸB in cancer is versatile, its suppression can affect several mechanisms beyond ferroptosis. The upregulation of SIRT6 can suppress pancreatic cancer through NF-ĸB’s nuclear transfer inhibition to impair glycolysis and facilitate ferroptosis [135]. However, a significant limitation of current studies is the lack of understanding of the crosstalk between autophagy and ferroptosis in the context of NF-ĸB. Since NF-ĸB regulates autophagy and from the view that ferroptosis is controlled by autophagy, it is crucial to explore the potential role of NF-ĸB in the regulation of autophagy and ferroptosis crosstalk.

### NF-ĸB and anoikis

When the cells are not attached to the extracellular matrix (ECM), or lack cell adhesion to the correct regions, they undergo a kind of cell death known as anoikis. The integrin receptors are responsible for cell-ECM interaction, and in addition to providing a connection with the cytoskeleton, they transduce the signals from ECM to cells [136]. The first description of anoikis was provided in epithelial and endothelial cells [137] and it is vital for development and tissue homeostasis. NF-κB is a regulator of anoikis in the cancer cells. For the process of tumorigenesis, the cancer cells use NF-κB as an alternative mechanism for causing resistance to anoikis. Gastric cancer progression and lymph node metastasis increase by DBC1. The stimulation of the IKK-β/NF-κB axis by DBC1 causes anoikis resistance in gastric tumors [138]. An identical process has also been followed in breast cancer in which DBC1 induces the IKK-β/NF-κB axis for the anoikis resistance [139]. After the loss of attachment, stimulation and nuclear translocation of NF-κB can increase levels of TrkB and NTF3. However, miR-200c downregulates ZEB1, TrkB, and NTF3 to increase anoikis sensitivity in triple-negative breast cancer [140]. Therefore, NF-κB transcription is vital for anoikis resistance. In esophageal cancer, NF-κB promotes PLK1 expression to suppress the degradation of β-catenin in the development of anoikis resistance [141].

### NF-κB and mitochondrial dynamics

The theory of mitochondrial origin, proposed by Lynn Margulis, is rooted in the endosymbiotic theory. In this mutualistic relationship, one bacterium entered the host cell, laying the foundation for the origin of mitochondria [142]. Mitochondria are present in almost all mammalian cells, and their role in the imbalance of mitochondrial quality during the development of tumors has been consistently emphasized [143, 144]. Increasing evidence suggests that tumor cells can gain advantages in proliferation and survival by modifying mitochondrial morphology and dynamics. Mitochondrial fission is prevalent in various types of tumors, including melanoma, ovarian, breast, lung, thyroid, and glioblastoma. Increased mitochondrial fusion is directly linked to chemotherapy resistance in tumor cells [145].

In recent years, researchers have delved into the intricate interplay between NF-κB and mitochondrial dynamics in the context of tumor development. Mutations in oncogenes, tumor suppressor genes, and metabolic enzymes result in significant alterations in multiple mitochondrial metabolic pathways, including oxidative phosphorylation, fatty acid, glutamine, and one-carbon metabolism, contributing to the theoretical foundation of the Warburg effect in tumor metabolism [146]. The intricate interplay between NF-κB and mitochondrial dynamics provides a novel perspective for a deeper understanding of their roles in cancer. Firstly, the activity of NF-κB is regulated by the oxidative stress status of mitochondria. Oxidative stress typically induces the generation of ROS within mitochondria, thereby influencing the activity of NF-κB. Conversely, the activation of NF-κB may modulate the level of mitochondrial ROS by regulating the expression of antioxidant genes. This reciprocal regulation mechanism is likely to play a crucial role in the survival and proliferation of tumor cells [147]. Secondly, the excessive activation of NF-κB can lead to alterations in mitochondrial morphology, resulting in an increase in mitochondrial fission and a decrease in fusion. This, in turn, affects the permeability and functionality of the mitochondrial membrane [148]. In the context of cancer, these NF-κB-induced changes in mitochondrial dynamics may represent one mechanism by which tumor cells escape apoptosis and enhance cell survival. Exploring the crosstalk between NF-κB and mitochondrial dynamics with other crucial cellular signaling pathways will contribute to a comprehensive understanding of the multifaceted factors influencing tumor development, laying the theoretical foundation for multi-targeted combination therapies.

### NF-ĸB and glycolysis

Six distinct hallmarks have been considered for cancer, including abnormal growth, evading proliferation inhibition, cell death resistance, angiogenesis, metastasis, and replicative immortality [149]. According to recent advances, metabolic reprogramming has been considered as another hallmark of cancer [150, 151]. Otto Warburg, in the 1920s, demonstrated that normal cells utilize oxidative phosphorylation in mitochondria for glucose catabolism, while tumor cells transform glucose into lactate for their metabolism [152]. This is known as aerobic glycolysis, as it is performed in high oxygen levels. The features of glycolysis include an increase in glucose uptake and lactate production. In spite of low ATP production in glycolysis, up to 50–70% of ATP for cancer cells is provided by glycolysis [153]. Recently, the regulatory pathways of glycolysis have been of importance, and NF-κB is among them. The proliferation of pancreatic cancer relies on glycolysis induction. The overexpression of NF-κB by IRAK2 can cause glycolysis-induced growth in pancreatic cancer. The application of maslinic acid as an NF-κB suppressor can impair the cancer progression [154]. For stimulation of glycolysis in cancer, NF-κB promotes c-Myc expression. Therefore, the utilization of compounds suppressing the NF-κ/c-Myc axis can suppress glycolysis. Betulinic acid is able to enhance caveolin-1 expression in inhibition of NF-κB/c-Myc axis to impair glycolysis in breast cancer [155]. Even the uptake of glucose in tumor cells can be regulated by the NF-κB axis. The low expression level of OVOL2 in lung cancer can increase tumorigenesis, while upregulated OVOL2 reduces the survival of cancer cells. Upon the attachment of OVOL2 to p65, it prevents the recruitment of P300 while it accelerates the interaction and binding of HDAC1 to p65 to suppress the NF-κB axis. Then, downregulation of GLUT1 occurs to reduce the glucose uptake in cancer cells [156].

### NF-ĸB and angiogenesis

**Fig. 5:**
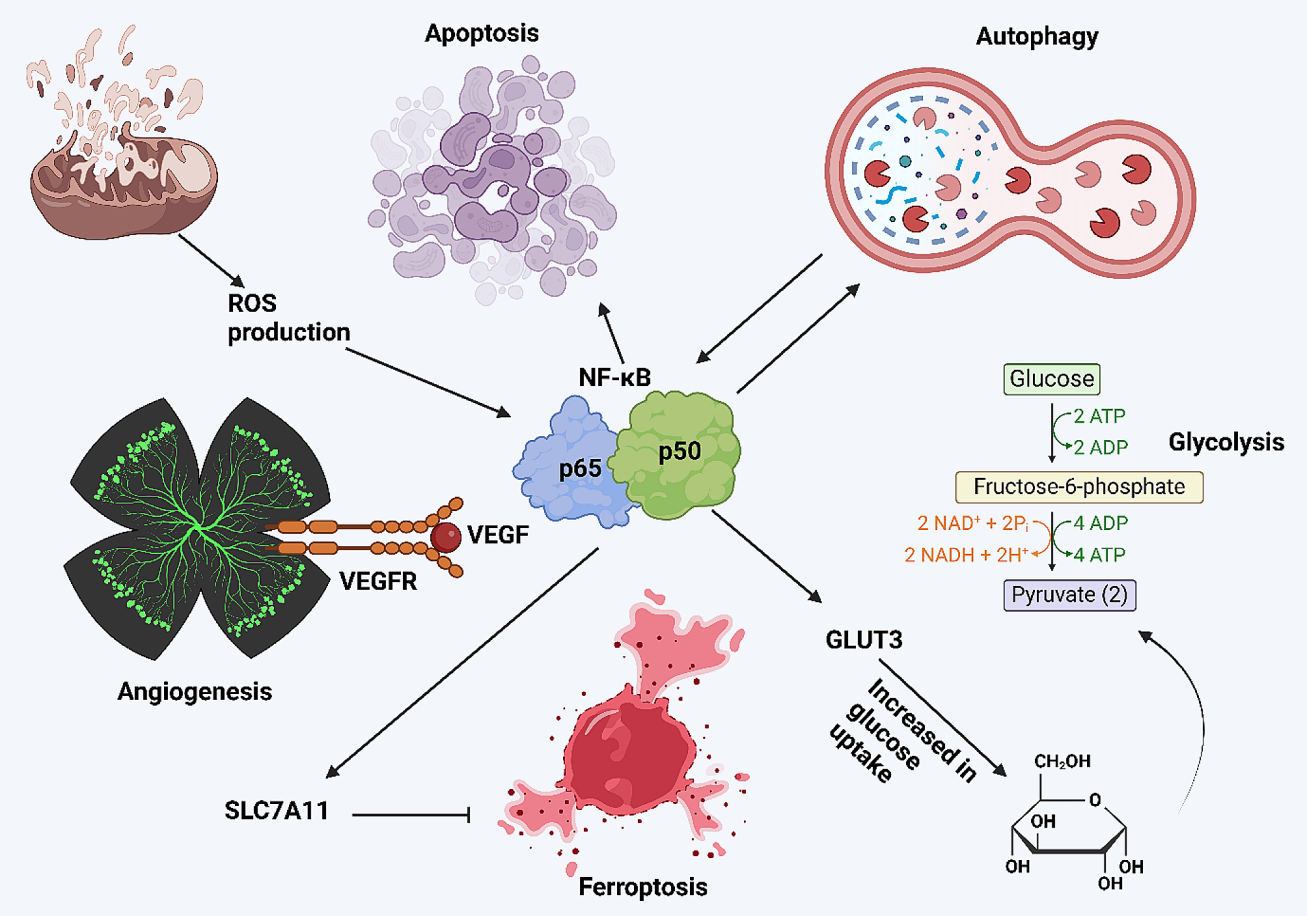
The NF-κB interaction with other biological mechanisms. The interesting part is the induction of apoptosis by NF-κB when the ROS levels in the mitochondria increase. Moreover, NF-κB promotes levels of VEGF to induce angiogenesis. The upregulation of SLC7A11 by NF-κB can disrupt ferroptosis. Furthermore, GLUT3 upregulation by NF-κB increases the glucose uptake in cancer cells to induce glycolysis. The interaction of autophagy and NF-κB is mutual, and in addition to NF-κB function in the regulation of autophagy, the autophagy mechanisms can also regulate NF-κB by degradation of related proteins (Created by Biorender.com)

Adequate oxygen and nutrients are vital for the tumor cells to survive and proliferate. Therefore, it is a necessity for the cancer cells to reside near the blood vessels [157]. After the observation that the cancer site demonstrates high vascularization, Judah Folkman suggested that angiogenesis is utilized for carcinogenesis [158]. After that, Folkman isolated a protein vital for angiogenesis induction [159]. Currently, the increasing evidence demonstrates the role of angiogenesis in cancer and its control by NF-κB. The upregulation of VEGF is vital for angiogenesis induction, and it is overexpressed by NF-κB [160]. However, NF-κB uses an indirect way to increase VEGF expression and its expression increases by the interactions between pathways and receptors. The presence of CXCL5 and its binding to the receptors on the surface of colorectal cancer cells causes the induction of the PI3K/Akt axis. Then, Akt induces nuclear translocation of p50/p65 to upregulate FOXD1. After that, FOXD1 upregulates VEGF to induce angiogenesis [161]. The IKKs induce NF-κB axis, while IkBα suppresses NF-κB. The conversion of PIP2 to IP3 causes upregulation of DAG to increase PKC levels. Then, PKC promotes phosphorylation of IKK at Ser176/180 to induce interaction of PLCE1 with p50/p65 in increasing nuclear transfer of this complex. Then, the p50/p65 complex promotes levels of VEGF-C to induce angiogenesis. Moreover, PIP2 conversion to IP3 increases the degradation of IkBα through its phosphorylation on Ser32. Then, the p50/p65 complex transfers into the nucleus to increase VEGF-C-mediated angiogenesis [162]. The inhibition of angiogenesis, proliferation, and NF-κB/STAT3 axis in colorectal cancer by analapril can increase drug sensitivity [163]. The NF-κB interaction with major biological mechanisms has been summarized in Fig. 5.

## Carcinogenic functions of NF-ĸB

### NF-ĸB and cancer growth and progression

Abnormal growth is a distinct feature of tumor cells characterizing them from normal cells. The aberrant proliferation of cancer results from the significant changes in the genomic profile. According to the recent advances in biological and medical science, the upregulation of NF-κB has been a common feature among the tumor cells with a high proliferation rate. This is not specific to a cancer type, and in all cancer types, the upregulation of NF-κB has been considered as a factor in fuelling tumorigenesis. In the case of renal cancer, GYS1 interacts with RPS27A and forms a complex of GYS1/RPS27A that can increase the nuclear transfer of p65. Then, this axis promotes glycogen accumulation and also, enhances the proliferation of cancer cells [164]. Sometimes, a protein can increase the growth of tumor cells, but more than one molecular pathway regulates its expression. Such complicated pathways have been observed in breast cancer that IFNα promotes nuclear transfer of p65 to upregulate IFITM1. Moreover, IFNα upregulates STAT2/IRF9 complex and its transfer to the nucleus for overexpression of IFITM1. Then, overexpressed IFITM1 increases the growth of triple-negative breast cancer [165]. However, the function of NF-κB in increasing the growth of triple-negative breast cancer is not only related to the increase in the levels of proteins. Sometimes, the chemokines are secreted by NF-κB to accelerate growth. The phosphorylation of NF-κB/p65 protein increases at the Ser536 site by kinectin 1 to increase its nuclear transfer. Then, upregulation of CXCL8 occurs to promote the progression and proliferation of breast cancer [166]. Notably, there is TLR4 receptor on the surface of tumor cells. PTX3 can bind to TLR4 receptor to mediate phosphorylation of IRAK-1. As a result, upregulation of Akt and JNK occurs that can induce NF-κB and c-Jun, respectively in promoting progression of tumor cells [167].

The presence of cancer stem cells (CSCs) in the colonies of tumor cells is not only a threat to the treatment of cancer but also causes the recurrence of cancer. The CSCs have self-renewal ability, and they can differentiate into other cells. The regulation of CSCs by NF-κB axis can provide new insights in the treatment of cancer. Targeting the NF-κB axis can affect the number and survival of CSCs as well as their differentiation. 5- fluorouracil is utilized for the treatment of hepatocellular carcinoma, but its combination with arsenic trioxide can cause a synergistic impact in tumor suppression. The attachment of LIF to the JAK1 receptors on the surface of cancer cells can increase STAT3 and NF-κB expression. A combination of arsenic trioxide and 5-fluorouracil can suppress LIF, STAT3, and NF-κB to stimulate the differentiation of CSCs [168]. The suppression of CSC properties in cancer upon NF-κB downregulation can be documented upon downregulation of ALDH1 and CD133 [169]. In order to improve stemness and CSC features, the PI3K/Akt axis is required for the regulation of NF-κB. C1ql4 induces the PI3K/Akt axis to upregulate NF-κB expression, inducing EMT and causing stemness in breast tumors [170]. The NF-κB axis induction can cause the secretion of a number of proteins in the stemness acceleration. When ROS levels are high in tumor cells, MT1G increases TRAF7 expression to suppress the NF-κB axis by enhancing p65 degradation. However, in low ROS levels, NF-κB induces secretion of Activin A from tumor cells to bind and induce ALK4/ActRII. Then, it promotes levels of Smad, Akt, and β-catenin in facilitating the CSC features in tumor cells [171].

### NF-ĸB and cancer metastasis

Metastasis is the major reason for the reduction in the survival rate of cancer patients [172]. Accumulating evidence highlights that the 5-year survival rate for metastatic cancer patients is lower than for localized cancer [173, 174]. Moreover, cancer invasion and metastasis can cause 90% of deaths in patients [175]. After the malignant transformation of normal cells and their aberrant proliferation and evasion from the immune system, these cells induce angiogenesis and achieve the invasive property. Then, they circulate and survive in blood and establish new colonies in distant tissues [176, 177]. Hence, major mechanisms in the metastasis of cancer should be highlighted, and NF-κB is among them. In several cancer types, the stimulation of NF-κB has been proposed as a factor in increasing invasive and aggressiveness potentials. NF-κB upregulation is dependent on PAR1 action, and moreover, suppression of MALT1 by siRNA impairs PAR1/NF-κB-mediated cancer metastasis. Furthermore, upon upregulation of NF-κB, a number of factors, including MMP-2 and cytokines such as IL-1β and IL-8, demonstrate upregulation, and their function in improving cancer invasion and progression can be explored [178].

**Fig. 6:**
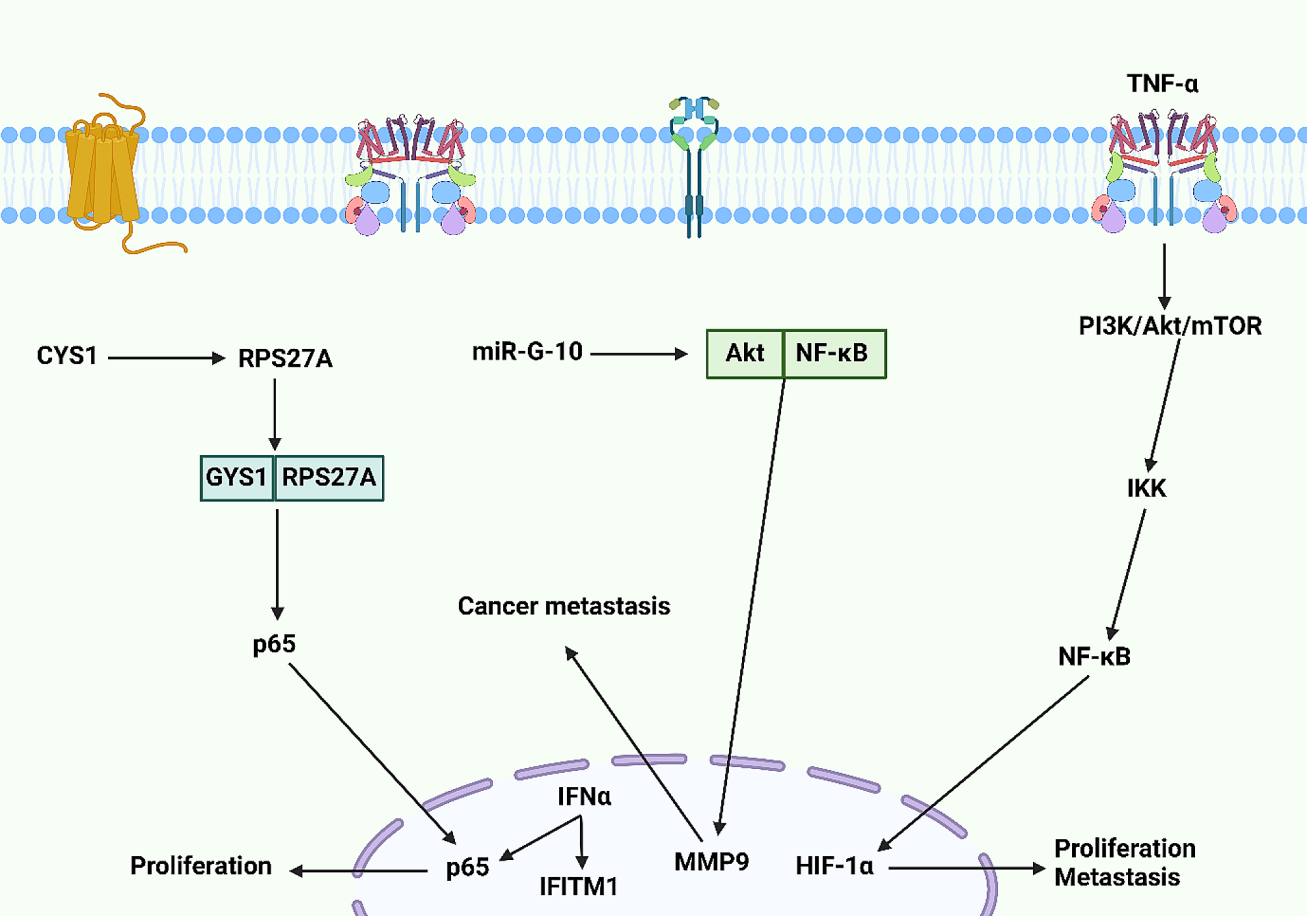
The involvement of NF-κB axis in the proliferation and invasion of cancer. NF-κB increases both proliferation and metastasis after transfer into the nucleus. CYS1 interacts with RPS27A to develop CYS1/RPS27A complex. Then, this complex promotes p65 expression and its nuclear transfer to enhance the proliferation. Moreover, IFNα promotes IFITM1 expression to facilitate the proliferation of cancer. The upregulation of MMP9 induced by the Akt/NF-κB axis can enhance the metastasis and invasion of cancer. Furthermore, TNF-α stimulates IKK through the upregulation of PI3K/Akt/mTOR to mediate the NF-κB axis. At the next step, NF-κB promotes HIF-1α expression to facilitate proliferation and invasion of cancer cells (Created by Biorender.com)

According to the previous discussions, the upregulation of MMP9, as a member of the matrix metalloproteinase family, can cause carcinogenesis. Without a doubt, epithelial-mesenchymal transition (EMT) is another player in cancer metastasis and therapy resistance [179, 180]. The transformations and alterations mediated by EMT can increase the migration and metastasis, and help in the development of secondary tumors [181, 182]. As a dynamic process, EMT results from the interactions and networks of growth factors, proteins, transcription factors, and molecular pathways [183]. Owing to the function of NF-κB in the regulation of cancer metastasis, there have been hypotheses regarding the regulation of EMT by NF-κB in cancer metastasis and invasion. The TNF-α insult in cancer can induce EMT. TNF-α promotes IKK expression to induce NF-κB axis. Moreover, TNF-α stimulates the PI3K/Akt/mTOR axis. Both mTOR and NF-κB participate in the upregulation of HIF-1α, and three distinct pathways are followed from here to increase the metastasis and migration of cancer cells. In the first step, HIF-1α promotes VEGF levels to increase angiostatin levels. Moreover, HIF-1α upregulates MMP-9 expression to increase invasion. Finally, HIF-1α promotes Snail levels to induce EMT in cancer invasion and metastasis [184]. Table 1 is a summary of NF-κB function in cancer metastasis. Figure 6 is an overview of the role of NF-κB axis in the regulation of cancer proliferation and metastasis.

**Table 1:** NF-κB fuels cancer metastasis and invasion

Cancer	Molecular targets	Remarks	Refs
Gastric cancer	NETO2/Akt/NF-κB	NETO2 stimulates PI3K/Akt axis to increase NF-κB expression Upregulation of Snail in the stimulation of cancer metastasis	[185]
Gastric cancer	PI3K/AKT/NF-κB/ZEB	Stimulation of PI3K/AKT/NF-κB/ZEB axis by BAG4 in cancer invasion and metastasis	[186]
Lung cancer	NF-κB	G9a stimulates the NF-κB axis to promote focal adhesion kinase induction	[187]
Colorectal cancer	NF-κB	FAP attaches to Enolase1 in NF-κB induction and acceleration of invasion and metastasis	[188]
Lung cancer	Tac2-N/NF-κB	Tac2-N stimulates the NF-κB axis in increasing cancer invasion and migration	[189]
Ovarian cancer	STAT3/AKT/NF-κB/IL-8	Ascites-derived ALDH + CD44 + tumour cell subsets increase PDK4 expression to induce STAT3/AKT/NF-κB/IL-8 axis in cancer invasion	[190]
Colorectal cancer	MGP/NF-κB	MGP induces the NF-κB axis to stimulate the exhaustion of CD8 + T cells in enhancing liver metastasis of colorectal tumor	[191]
Breast cancer	CircIKBKB/NF-κB	Upregulation of circIKBKB promotes NF-κB expression to induce bone metastasis of breast cancer CircIKBKB expression increases by the function of EIF4A3	[192]
Prostate cancer	NF-κB/Activin A	NF-κB promotes Activin A expression to induce metastasis and increase CSC-like subpopulation	[193]
Lung cancer	MEST/NF-κB	MEST interacts with VCP to induce the NF-κB axis for metastasis	[194]
Bladder cancer	ROC1/NF-κB	ROC1 induces NF-κB axis for increasing lymph node metastasis and progression	[195]
Gastric cancer	UBAP2L/NF-κB	UBAPL2 promotes PI3K/Akt expression to induce the NF-κB axis	[196]
Breast cancer	NF-κB	Conversion of palmitate to acetyl-CoA in breast cancer cells Palmitate increases expression of lysin acetyltransferase 2 A to induce acetylation of NF-κB in metastasis formation	[197]

### NF-ĸB and cancer drug resistance

Cancer drug resistance has appeared as a challenge for healthcare physicians dealing with cancer therapy and management. Cancer drug resistance can be divided into two parts, including acquired and intrinsic resistance; in which acquired drug resistance occurs upon exposure to chemotherapy drug and subsequent alterations in the tumor cells, while intrinsic drug resistance is a result of genetic and epigenetic changes before chemotherapy drug exposure. NF-κB axis has been considered a regulator of drug resistance in human cancers. The biological studies have highlighted the interaction of NF-κB with other molecular pathways in the regulation of cancer progression. NF-κB mainly participates in intrinsic drug resistance, but there is also evidence showing that exposure to chemotherapy drugs can activate the NF-κB axis for resistance development. Although efforts have revealed the complicated mechanisms involving NF-κB in the process of drug resistance, it should be evaluated if these mechanisms are “druggable targets” or not. Moreover, in case of being a “druggable target”, the investigation should be performed on the efficacy of targeting these mechanisms for reversing drug resistance. The mission of this section is to evaluate the role of NF-κB axis in the regulation of cancer drug resistance. The utilization of other anticancer drugs can increase the sensitivity to chemotherapy. Ivermectin is considered as a factor for increasing drug sensitivity. Ivermectin reduces EGFR expression to suppress the ERK/Akt axis. Then, NF-κB downregulation occurs to reduce P-gp function and activity in overcoming chemoresistance [198]. Another drug used for the combination of cancer therapy and reversing resistance is fatostatin. In endometrial carcinoma, fatostatin downregulates SREBP1 to suppress the NF-κB axis for inducing apoptosis and reversing progesterone resistance as a therapeutic strategy [199]. Even histone deacetylases demonstrate interaction with NF-κB in the regulation of cancer drug resistance. The interaction of NF-κB and histone deacetylases is vital for the tumorigenesis. The expression of HDAC5 increases by CD13, and then it stimulates the NF-κB axis through the upregulation of LSD1 to cause sorafenib resistance in hepatocellular carcinoma [200]. However, NF-κB is not the only executor in the process of chemoresistance. TGF-β2 stimulates the EMT mechanism, and it induces nuclear translocation of p65 for inducing osimertinib resistance [201]. The newly developed genetic tools also participate in reversing cancer chemoresistance. It has been well-documented that the CRISPR/Cas9 system and screening can help us understand the main mechanisms that contribute to the process of drug resistance. Through the application of the CRISPR/Cas9 system, it was found that Akt stimulates the NF-κB axis to upregulate E2F6 expression in the development of temozolomide resistance [202]. Table 2 summarizes the role of NF-κB in the development of cancer drug resistance.

**Table 2:** NF-κB and cancer resistance in crosstalk

Cancer	Molecular interaction	Remark	Ref
Breast cancer	TRIM47/NF-κB	TRIM47 stabilizes PKC-ε/PKD3 to induce NF-κB axis for endocrine resistance development	[203]
Oral cancer	LCN2/NF-κB	Vitamin D enhances response to cisplatin chemotherapy through suppression of LCN2-mediated NF-κB	[204]
Colorectal cancer	CD133/NF-κB	CD133 stimulates the NF-κB axis through Akt upregulation to induce MDR1	[205]
Prostate cancer	Caspase-8/NF-κB	Caspase-8 stimulates NF-κB axis to induce enzalutamide resistance	[206]
Prostate cancer	NF-κB	Suppression of NF-κB by DMAPT drug can impair resistance to AR inhibition	[207]
Breast cancer	HSPB1/NF-κB	HSPB1 stimulates NF-κB to induce drug resistance	[208]
Lung cancer	CHD1L	CHD1L stimulates c-Jun expression to upregulate ABCB1 in the stimulation of the NF-κB axis for cisplatin resistance	[185]
Melanoma	NF-κB	The suppression of NF-κB increases BET inhibition sensitivity	[209]
Breast cancer	miR-34a	Combination therapy of doxorubicin and miR-34a can suppress the Notch/NF-κB axis in accelerating drug sensitivity	[210]
Breast cancer	NF-κB	The function of NF-κB upregulation in the development of tamoxifen resistance	[211]
Glioma	NF-κB/STAT3	Upregulation of the NF-κB/STAT3 axis induces resistance to Smac mimetics	[212]
Lung cancer	miR-146b-5p	miR-146b-5p increases drug sensitivity through suppression of IRAK1/NF-κB axis	[213]
Prostate cancer	NF-κB/IL-6/STAT3	Gut microbiota increases NF-κB expression to induce IL-6/STAT3 axis in docetaxel resistance development	[214]

### NF-ĸB and cancer radioresistance

**Fig. 7:**
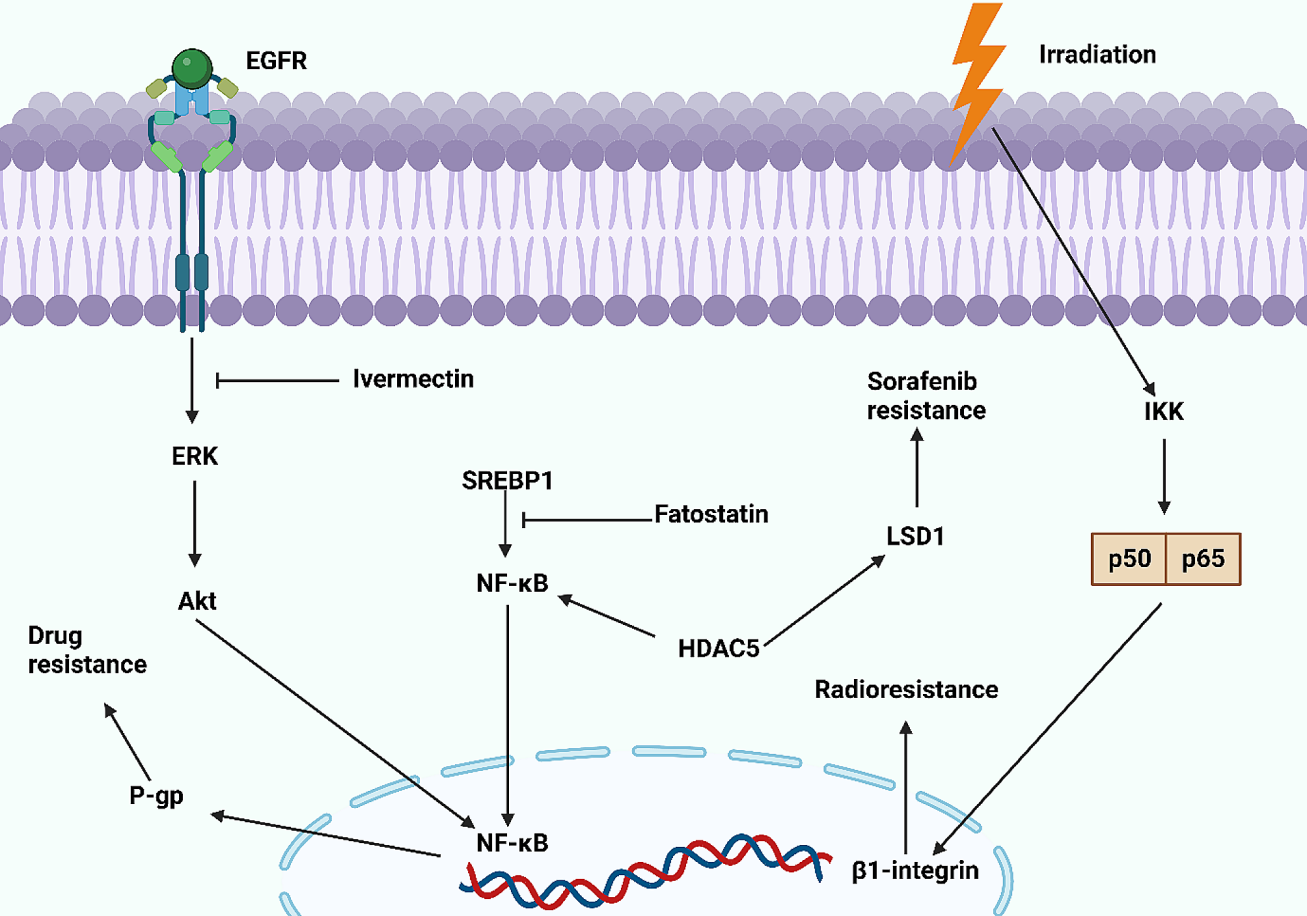
The NF-κB axis participates in the development of chemoresistance and radioresistance in cancer. The stimulation of EGFR triggers the ERK/Akt axis to mediate the nuclear transfer of NF-κB. Then, NF-κB increases P-gp expression to induce drug resistance. Furthermore, SREBP1 stimulates NF-κB to prevent apoptosis in the development of drug resistance, while the SREBP1/NF-κB axis is disrupted by fatostatin. Moreover, HDAC5 positively interacts with NF-κB to increase LSD1 expression in sorafenib resistance. The exposure of cancer cells to irradiation increases IKK expression to mediate β1-integrin expression through the nuclear transfer of the p50/p65 complex to induce radioresistance (Created by Biorender.com)

Various kinds of therapeutic modalities have been applied for cancer, ranging from surgical resection, immunotherapy, targeted therapy, chemotherapy, and radiotherapy. Regarding the sensitivity of tumor cells to radiotherapy, irradiation has emerged as a potential strategy for cancer elimination [215–217]. In radiotherapy, two distinct ways are utilized for cancer suppression, including stimulation of DNA damage and an increase in ROS production [218]. Moreover, the combination of radiotherapy with immunotherapy and chemotherapy can accelerate tumor suppression through impairing hypoxia by decreasing oxygen consumption by cancer cells and changing the immune response, leading to improvements in clinical outcomes [219, 220]. ALDH1L1 is an essential enzyme of folate metabolism whose function is tumor-suppression, and it is a reliable biomarker for cancers [221, 222]. The mitochondrial homolog of ALDH1L1 is known as ALDH1L2, and it is a product of a distinct gene on chromosome 12q23.3 [223]. The ALDH1L1 and ALDH1L2 share some structural and functional similarities, and they are capable of catalysing 10-fTHF hydrolase and 10-fTHF dehydrogenase reactions [223–225]. ALDH1L2 is considered as an irradiation-related factor and reduction in its expression can cause resistance through suppression of ROS-induced apoptosis. However, for stimulation of radioresistance, ALDH1L2 is not alone, and through interaction with TXN, it induces the nuclear transfer of NF-κB, upregulating SOD2 and CAT levels to reduce ROS generation for radioresistance development [226]. As it was mentioned, ALDH1L1 is related to the folate metabolism. Therefore, it can be concluded that metabolism and metabolic factors can participate in the development of radioresistance in human cancers. To evaluate such a hypothesis, the role of fatty acids in the regulation of radioresistance in prostate cancer has been investigated. The suppression of fatty acid can enhance the radiosensitivity in prostate cancer. Moreover, the combination of radiotherapy and orlistat results in the suppression of NF-κB and related proteins such as FASN to increase radiosensitivity [227]. Hence, the combination therapy for NF-κB suppression appears to be a promising strategy for increasing radiosensitivity in cancer (Fig. 7).

## Epigenetic regulation of NF-κB by non-coding RNAs

**Fig. 8:**
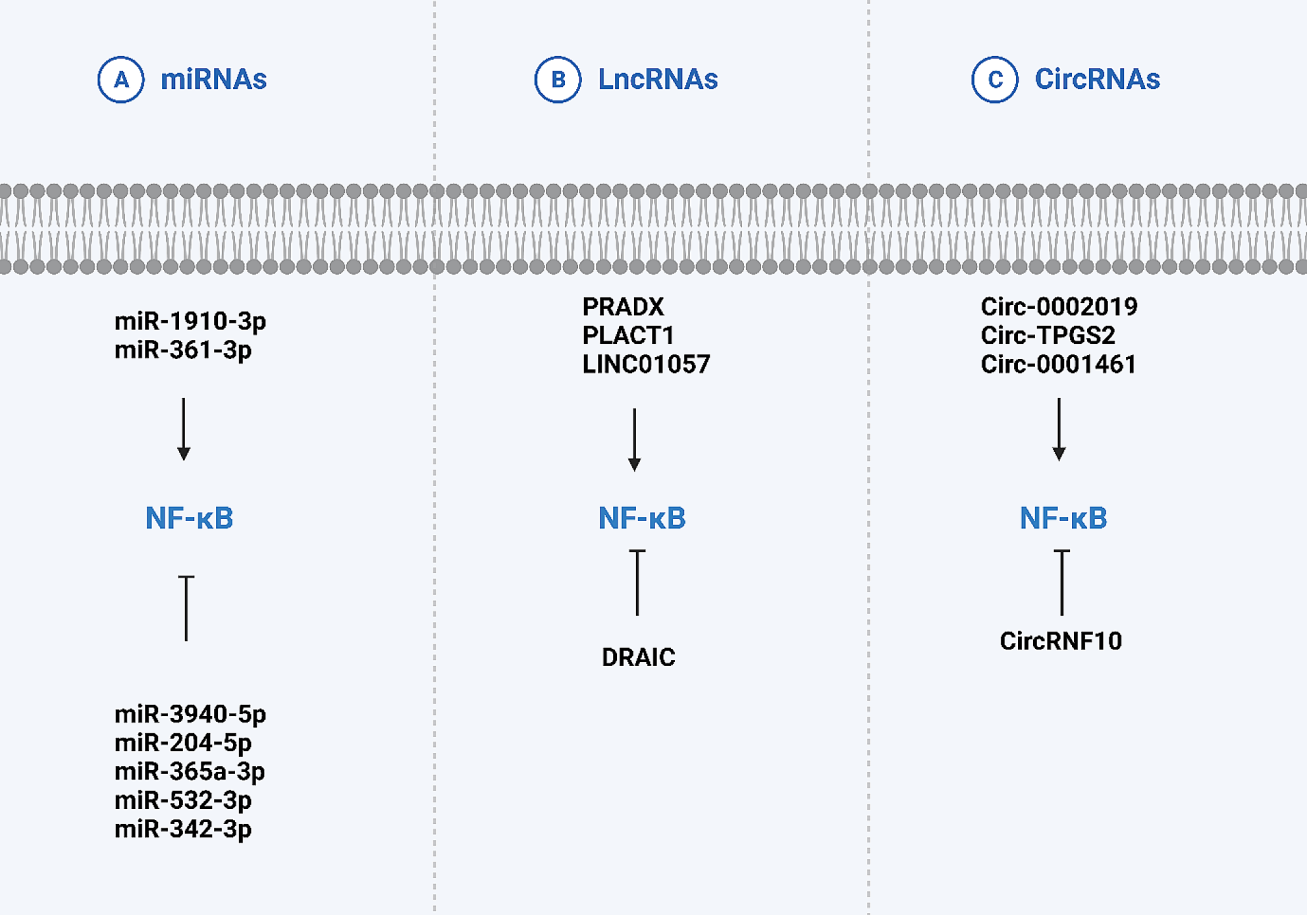
The NF-κB regulation by non-coding RNAs in cancer. Although the studies have highlighted the role of these RNA molecules in NF-κB control, there is another side which is the regulation of RNAs by NF-κB such as an increase in miR-574 expression by NF-κB (Created by Biorender.com)

The role of non-coding RNAs in the regulation of cancer progression has been evaluated extensively. However, since a high number of RNAs have been identified in recent years, the current ideas should be directed toward specific targets of non-coding RNAs in cancer. The major non-coding RNAs can be categorized into miRNAs, lncRNAs, and circRNAs, with the potential for cancer progression and drug resistance regulation. Recently, it has been shown that non-coding RNAs are major modulators of the NF-κB axis in cancer, and this can change the proliferation, metastasis, and therapy resistance of tumor cells. As single-stranded RNA molecules, miRNAs are able to bind to 3’-UTR of targets in their suppression. Therefore, NF-κB should have binding sites for specific miRNAs to regulate its expression. This expression modulation can occur at the mRNA and protein levels and finally, it affects carcinogenesis. The mature and functional miRNAs are found in the cytoplasm. Therefore, they are not able to affect nuclear transferred NF-κB. The IL-6 binds to IL-6R on the receptor of cancer cells to induce STAT3 axis. Then, STAT3 is transferred into the nucleus to increase miR-135b biogenesis. Then, its mature type is formed in the cytoplasm, and miR-135b stimulates NF-κB. The presence of CYLD can suppress NEMO complexed with IKKs. However, miR-135b reduces CYLD expression to induce NF-κB axis through increasing proteasomal degradation of IκBα. Then, the p50/p65 complex transfers into the nucleus to enhance levels of IL-6, IL-8, Bcl-2, Bcl-xL, MMPs, and CCND1 in increasing cancer progression and reducing apoptosis [228]. NF-κB protein is implicated in promoting cell growth and developing drug resistance. TRAF6 activates the NF-κB pathway, thereby facilitating tumorigenesis. Conversely, miR-146a-5p suppresses NF-κB activity by downregulating TRAF6. This, in turn, leads to the downregulation of P-gp, impairing drug resistance and reducing the proliferation of cancer cells [229]. The TME remodeling and the change in the cancer cells from primary to metastatic can be induced by miRNA/NF-κB interaction. The increase in the biogenesis of miR-192-5p can cause the development of mature miR-192-5p in the cytoplasm. Then, miR-192-5p downregulates RB1 expression to induce NF-κB p65 axis. Then, secretion of IL-10 occurs to bind to IL-10R in the stimulation of Foxp3 + Treg cells. Moreover, this axis stimulates EMT and increases the growth and metastasis of cancer cells [230]. The positive point of studies is the regulation of the NF-κB axis by lncRNAs in cancer. Since lncRNAs have a linear structure and binding sites, the NF-κB proteins are able to bind to the promoter of lncRNAs for the regulation of their expression. On the other hand, lncRNAs can modulate miRNA expression by sponging. LncRNA AC007271.3 expression is enhanced by NF-κB as an attempt to increase cancer progression. The NF-κB/AC007271.3 axis destabilizes miR-125b-2 to relieve its inhibitory impact on Slug. Then, overexpressed Slug increases N-cadherin, vimentin, and β-catenin levels while it reduces N-cadherin levels to mediate metastasis of oral cancer [231]. However, miRNAs are not the only targets of lncRNAs. Moreover, the lncRNAs can modulate NF-κB expression. The presence of PHLPP can suppress Akt and IKKα in tumor suppression. Moreover, when androgen deprivation therapy occurs, the prostate tumor cells make changes, displacing PHLPP from the IKKα/FKBPS1 complex. Then, lncRNA PCAT1 stimulates the NF-κB axis by IKKα expression. Furthermore, lncRNA PCAT1 stimulates the PI3K/Akt axis to enhance tumorigenesis [232]. As a result, NF-κB and lncRNAs have mutual functions and can regulate the expression level of each other. Another factor in the regulation of the NF-κB axis is circRNAs with a special structure (covalently closed loop structure). Since circRNAs are new emerging factors in cancer progression, a few studies have focused on NF-κB regulation by these RNA molecules. Notably, the current idea is that circRNAs regulate the nuclear transfer of NF-κB in the regulation of cancer progression. The interaction of p65 with DHX15 in the formation of a complex can mediate the nuclear transfer of p65. Moreover, p65 enhances the transcription of DHX15 in the nucleus to increase its levels. However, circRNF10 suppresses the DHX15/p65 complex to impair its nuclear transfer and suppress the NF-κB axis [233]. Table 3 and Fig. 8 summarize the role of non-coding RNA transcripts in the regulation of NF-κB axis.

**Table 3:** The non-coding RNA transcription regulation of NF-κB

Cancer	Non-coding RNA	Remark	Ref
Breast cancer	miR-1910-3p	The miR-1910-3p enriched in exosomes can stimulate the NF-κB axis to increase autophagy, growth, and invasion	[234]
Glioma	miR-3940-5p	miR-3940-5p downregulates CUL7 expression to suppress NF-κB	[235]
Thyroid carcinoma	miR-574	NF-κB promotes miR-574 expression to suppress BNIP3	[236]
Prostate cancer	miR-204-5p	NF-κB suppression by miR-204-5p to disrupt bone metastasis	[237]
Pancreatic cancer	miR-365a-3p	Suppression of c-Rel-induced NF-κB axis	[238]
Prostate cancer	miR-532-3p	miR-532-3p suppresses the NF-κB axis to disrupt bone metastasis	[239]
Pancreatic cancer	miR-342-3p	miR-342-3p suppresses dysbindin in the cytoplasm to impair NF-κB/MDM2-mediated cancer invasion and metastasis	[240]
Colorectal cancer	miR-361-3p	miR-361-3p downregulates TRAF3 expression to increase NIK interaction with IKKα Then, the p100/RelB complex promotes RelB/p52 complex levels, and after nuclear transfer, they increase proliferation and suppress apoptosis	[241]
Glioblastoma	LncRNA PRADX	PRADX recruits the PRC2/DDX5 axis to impair UBXN1 expression and induce the NF-κB axis	[242]
Pancreatic cancer	LncRNA PLACT1	Positive feedback loop of PLACT1 and NF-κB axis in the stimulation of NF-κB	[243]
Glioblastoma	LINC01057	LINC01057 stimulates the NF-κB axis to facilitate mesenchymal differentiation	[244]
Prostate cancer	LncRNA DRAIC	LncRNA DRAIC negatively interacts with IKK to suppress NF-κB	[245]
Gastric cancer	Circ-0002019	Circ-0002019 stimulates the NF-κB axis through TNFAIP6 upregulation in accelerating growth and metastasis	[246]
Breast cancer	Circ-TPGS2	Circ-TPGS2 sponges miR-7 to induce TRAF6/NF-κB axis in causing dysregulation of TME	[247]
Oral cancer	Circ-0001461	Circ-0001461 sponges miR-145 to induce TLR4/NF-κB axis in cancer progression	[248]

## NF-ĸB inhibitors

### Natural compounds

The natural compounds have been used extensively for the regulation of NF-κB and its suppression in cancer therapy. There is a significant difference between phytochemicals and small molecule inhibitors in that phytochemicals have pleiotropic functions and regulate different pathways to finally inhibit NF-κB, while small molecule inhibitors specifically target certain proteins of the NF-κB axis. However, current experiments highlight the role of plant-derived natural products as efficient compounds in NF-κB suppression. A limitation of phytochemicals is their poor bioavailability and therefore, their clinical application and therapeutic index are restricted. As a result, it is suggested to use nanoparticles for the targeted delivery of natural products to improve their potential in the regulation of NF-κB and cancer therapy. Solasodine is an example of a natural product that can impair the metastasis and invasion of gastric cancer through the regulation of NF-κB. Solasodine promotes levels of AMPK to suppress the NF-κB axis through STAT3 downregulation. Then, inhibition of claudin-2 occurs to reduce the invasion and migration of tumor cells [249]. This example demonstrates that natural compounds mainly target the regulated pathways of NF-κB instead of targeting the specific proteins of NF-κB. In addition to STAT3, HIF-1α can promote the expression of NF-κB to induce colorectal cancer invasion. The application of Calebin A, as a bioactive compound of turmeric, suppresses the HIF-1α/NF-κB axis, thereby impairing metastasis [250].

### Synthetic and small molecules

**Fig. 9:**
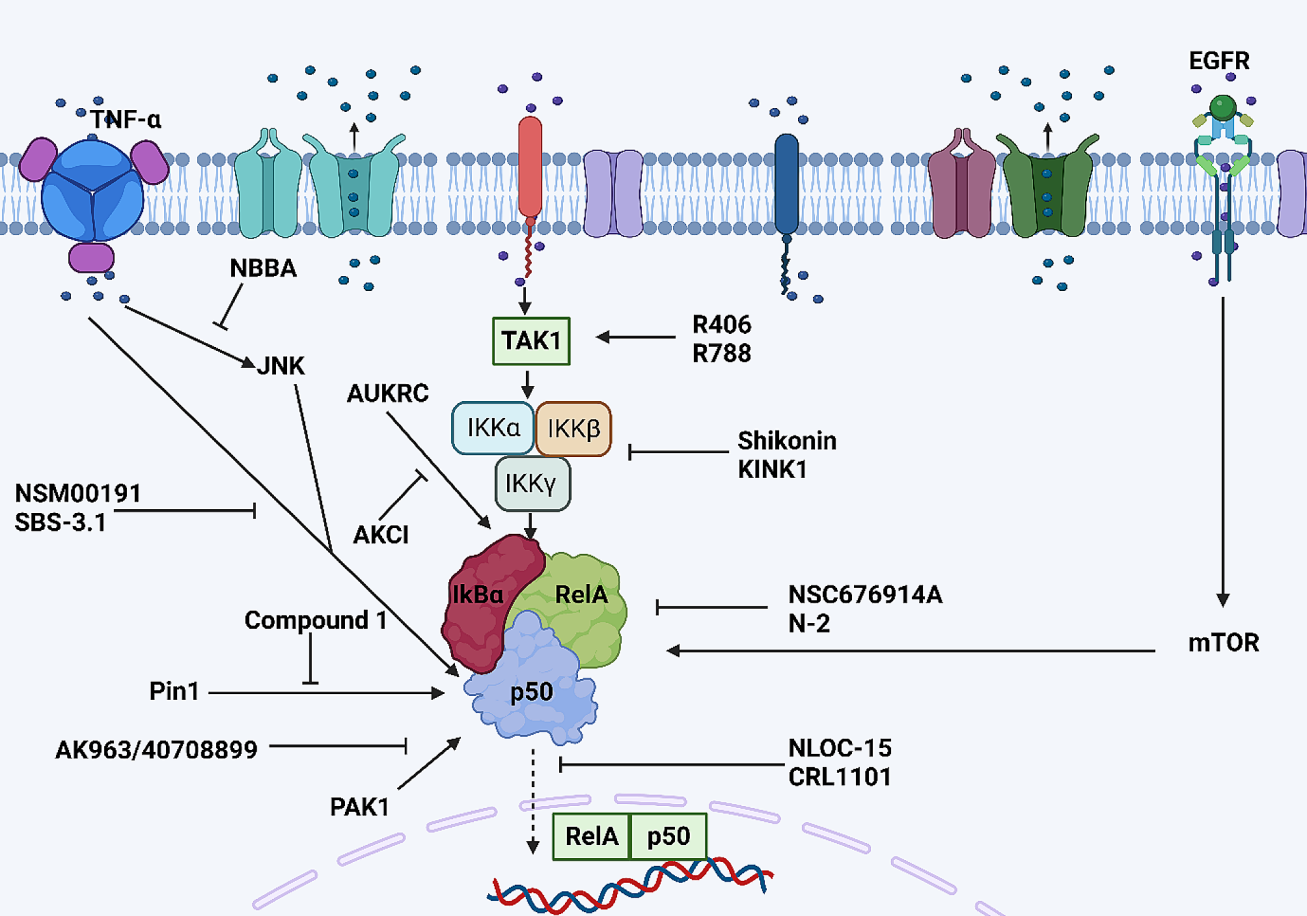
The small molecule inhibitors of NF-κB (Created by Biorender.com)

Since the several proteins related to NF-κB have been understood, the small molecules targeting such proteins have been introduced. The upregulation of IKKβ and its interaction with NEMO are vital for NF-κB induction. Shikonin, as a small molecule, has been introduced to suppress IKKβ for NF-κB downregulation and suppression of colorectal cancer proliferation [251]. The interaction of p65 (RelA) with Pin1 can induce the NF-κB axis in the progression of prostate cancer, while this interaction is suppressed by compound 1 [252]. The cytokines and growth factors have been considered as regulators of NF-κB. The TNF-α activates NF-κB, while NSM00191 suppresses the TNF-α/NF-κB axis [253]. Similar to NSM00191, SBS-3.1 suppresses TNF-α/NF-κB axis in the treatment of lung cancer [254]. Furthermore, TNF-α increases JNK expression to induce NF-κB activation, while NBBA suppresses TNF-α and JNK interaction, impairing the NF-κB axis [255]. Moreover, a novel inhibitor, NLOC-015 A reduced the oncogenic characteristics while concurrently decreasing the expression levels of EGFR, mTOR, AKT, and NF-κB signaling network in non-small-cell lung cancer cell lines [256]. Since IKKβ function is required for NF-κB axis induction, another small molecule inhibitor known as KINK-1 has been developed to suppress IKKβ in the treatment of melanoma [257]. Figure 9 demonstrates the small molecule inhibitors of the NF-κB axis. Table 4 summarizes the status of NF-ĸB in clinical trials.

**Table 4:** The NF-ĸB in clinical trials in cancer patients

Cancer type	Phase	Purposes	ClinicalTrials.gov ID
Breast cancer	Phase II	Use of curcumin for inhibiting the DNA binding activity of NF-κB and changing levels of IL-6 as a downstream target for breast cancer patients that have completed chemotherapy and are receiving XRD	NCT01740323
Solid tumors or melanoma	Phase I Phase II	Inhibition of NF-κB signaling in melanoma therapy In this study, PS-341 as a proteasome inhibitor is utilized in combination with temozolomide (oral administration) to treat patients with solid tumors or melanoma	NCT00512798
Multiple myeloma	Not applicable	The application of curcumin in combination with Bioperine in the treatment of patients Evaluating the expression levels of NF-κB and related genes	NCT00113841
Osteosarcoma	Observational	Evaluating the response of cancer patients to pre-operative chemotherapy Evaluating NF-κB expression to predict response to chemotherapy	NCT00686738
Acute myeloid leukemia	Phase II	Evaluating NF-κB expression, when cancer patients under chemotherapy Observing if salicylate can change the expression level of NF-κB	NCT02144675
Multiple myeloma	Phase II	Use of curcumin and potential of NF-κB inhibition	NCT01269203
Gastric cancer	Observational	The application of NF-κB/JNK as a biomarker in the clinical trial to predict the response of cancer patients to chemotherapy	NCT01905969

## Summary, conclusion and future perspectives

The regulation of the NF-ĸB axis has built the blocks for cancer therapy. The current status of NF-ĸB function in human cancers reveals its versatile function in tumorigenesis. However, there is a paradox in the function of NF-ĸB that sometimes, it may exert tumor-suppressor effects such as regulation of dendritic cell function. Therefore, the therapeutic targeting of NF-ĸB is mainly based on the suppression of this axis, and sometimes, according to its tumor-suppressor activity, the induction of this pathway can be followed. Both canonical and non-canonical pathways of NF-ĸB participate in the progression of cancer, and after nuclear transfer of NF-ĸB components, including RelA/p50 or RelB/p52, and owing to the DNA binding activity of these complexes, the expression level of target genes can be modulated to affect various hallmarks of cancer. The evaluation of NF-ĸB function in the regulation of biological mechanisms in cancer demonstrates that NF-ĸB shows interaction with apoptosis, autophagy, ferroptosis, and other mechanisms. The current studies provide both positive and negative points regarding these interactions that can be summarized. At first, NF-ĸB can induce or suppress apoptosis in cancer. The increase in ROS production in mitochondria stimulates the NF-ĸB/apoptosis axis. The interaction of NF-ĸB and autophagy is interesting. While NF-ĸB has been considered a regulator of autophagy, studies demonstrate that autophagy can induce the degradation of various NF-ĸB-related proteins to induce/suppress the NF-ĸB axis. However, the problem is that the function of autophagy is pro-survival or pro-death, and therefore, if its induction occurs by NF-ĸB, the exact function on tumor cells requires investigation. The upregulation of NF-ĸB suppresses ferroptosis in tumor cells mainly through overexpression of SLC7A11. Moreover, the anoikis resistance can be induced by NF-ĸB. Although studies have highlighted the function of NF-ĸB in the regulation of cell death mechanisms, a limitation is the lack of investigation of NF-ĸB interaction with immunogenic cell death in different tumors. The fuel for cancer progression is provided by glycolysis, and NF-ĸB stimulates glycolysis. However, a more comprehensive investigation of the role of NF-ĸB in the regulation of glycolysis-related enzymes, including HK2, LDHA, and others is required. Moreover, CSC features are improved by NF-ĸB in human cancers, and in order to enhance vascularization in tumor sites, NF-ĸB upregulates VEGF levels to induce angiogenesis.

The interaction of NF-ĸB with TME components can regulate the progression of cancer. NF-ĸB demonstrates interaction with TAMs, CAFs, dendritic cells, NK cells, and T cells. In the TAMs and CAFs, the upregulation of NF-ĸB can accelerate tumorigenesis. Moreover, NF-ĸB can induce CAFs to secret ILs, and furthermore, the ILs secreted by cancer cells can induce NF-ĸB in CAFs. Moreover, the upregulation of NF-ĸB in TAMs increases their M2 polarization in tumorigenesis. In DC cells, NF-ĸB promotes IRF1 expression to enhance anti-cancer immunotherapy. Moreover, TLR1-mediated NF-ĸB axis can induce DC cells in TME. The upregulation of NF-ĸB can increase MHC-I antigen presentation and recruitment of CD8 + T cells. Therefore, NF-ĸB potentially regulates the various components of TME. The regulatory function of NF-ĸB on the major hallmarks of cancer has been evaluated, showing that NF-ĸB can accelerate proliferation, metastasis, chemoresistance, and radioresistance. Moreover, NF-ĸB participates in the biochemical recurrence of cancer. Therefore, the therapeutic suppression of NF-ĸB can significantly diminish the progression of cancer. With respect to the potential of NF-ĸB in the process of tumorigenesis (although it has onco-suppressor function in some cases), the suppression of NF-ĸB using phytochemicals and small molecule inhibitors has been followed. The difference between these two groups of compounds is that phytochemicals have a pleiotropic function and regulate various molecular pathways to finally suppress NF-ĸB, while small molecule inhibitors target the specific proteins of NF-ĸB in its regulation. Although phytochemicals and small molecule inhibitors have high potential in the regulation and inhibition of the NF-ĸB axis, one of their major problems is the lack of appropriate pharmacokinetic profile and bioavailability. Therefore, the application of nanoplatforms for the delivery of such compounds can increase the potential for tumor suppression and NF-ĸB downregulation. One of the important aspects in the recent years is the introduction of nanoparticles for the cancer immunotherapy, chemotherapy and phototherapy [258–260]. Therefore, it is of high importance to exploit the role of nanoparticles in regulation of molecular pathways. Recently, nanoparticles have been introduced for targeted suppression of the NF-ĸB axis in cancer therapy [261, 262]. Therefore, the delivery of small molecule inhibitors and phytochemicals alone or in combination for cancer therapy and NF-ĸB regulation is suggested.

There have been investigations in clinical trials regarding the translation of current pre-clinical findings. According to the pre-clinical studies, the upregulation of NF-ĸB causes tumorigenesis. Therefore, its suppression can improve cancer elimination. The use of NF-ĸB in clinical trials has been performed mainly in two categories, including the use of NF-ĸB as a biomarker for the chemotherapy response of cancer patients before or after surgery. Moreover, a number of NF-ĸB inhibitors have been used along with chemotherapy to improve the potential for cancer suppression. Therefore, targeting NF-ĸB is emerging in clinical trials, and more studies should be performed to understand the role of NF-ĸB in radioresistance, and its association with lymph node metastasis in cancer patients. Although it is not related to the clinical trials, it is worth mentioning that pre-clinical studies should also focus on understanding the role of NF-ĸB in tumorigenesis in vitro using 3D culture to better understand the dynamics and associations with other networks. Moreover, since studies have evaluated the dysregulation of NF-ĸB in different cell lines of a cancer and abnormal expression of canonical and non-canonical pathways, clinical studies should also focus on the different expression levels of NF-ĸB in cancer patients.

## Data Availability

No datasets were generated or analysed during the current study.
